# Investigation of the Effectiveness of Photo Deprotection of Polypeptides in Solution and within the Core of Miniemulsion-Derived Nanoparticles

**DOI:** 10.1021/acs.macromol.3c02538

**Published:** 2024-02-27

**Authors:** Nicola Judge, Andreas Heise

**Affiliations:** † Department of Chemistry, RCSI University of Medicine and Health Sciences, Dublin D02 YN77, Ireland; ‡ Science Foundation Ireland (SFI) Centre for Research in Medical Devices (CURAM), RCSI, Dublin D02 YN77, Ireland; § AMBER, The SFI Advanced Materials and Bioengineering Research Centre, RCSI, Dublin D02 YN77, Ireland

## Abstract

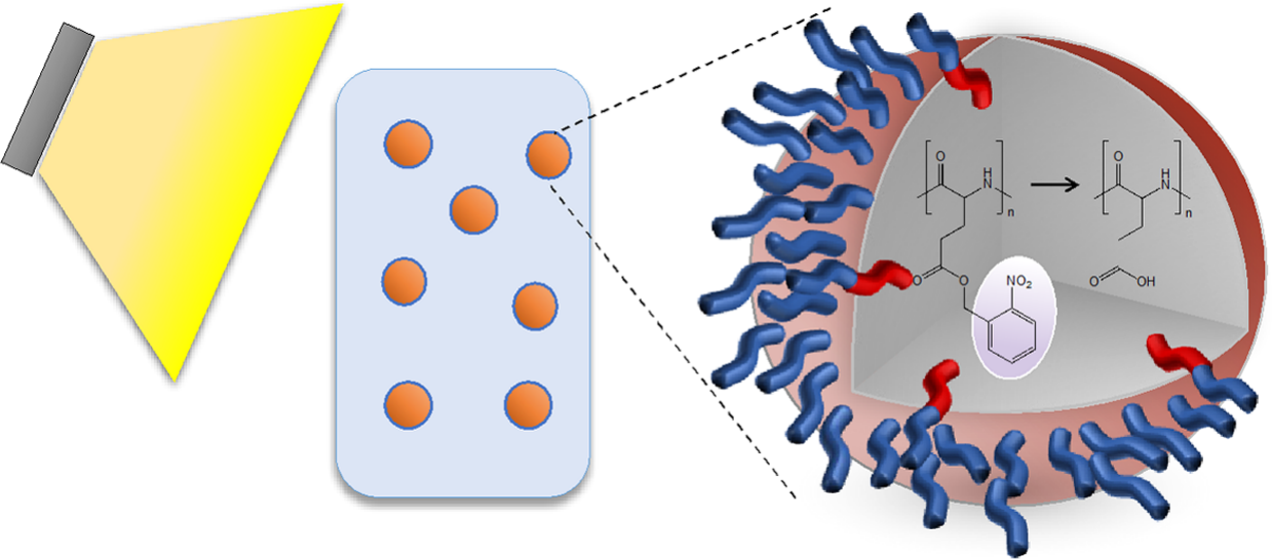

Homopolymerization of ortho-nitrobenzyl (*o*NB)-protected l-cysteine and l-glutamic acid was systematically studied in different solvents and at different monomer to initiator ratios, revealing the best reaction control in dimethylformamide (DMF) across a range of degrees of polymerization. In the subsequent ultraviolet (UV)-cleavage studies, it was found that quantitative deprotection upon UV exposure at 365 nm was not achievable for either of the homopolypeptides as confirmed by ^1^H NMR and UV/visible (UV/vis) analyses. While the poly(*o*NB-l-cysteine) deprotected more readily with no effect of the polypeptide molecular weight, lower molecular weight poly(*o*NB-l-glutamate) reached maximum deprotection faster than high molecular weight samples. This was further confirmed by the pH changes of the solution. When incorporated into the core of miniemulsion-derived nanoparticles, both *o*NB-protected copolypeptides were successfully deprotected as evident from a color change and a pH change in the case of poly(*o*NB-l-glutamate). However, the removal of the deprotection byproduct nitrosobenzaldehyde proved unsuccessful, which indicates a diffusion barrier caused by the nanoparticle’s surfactant. The study provides insights and guidelines for the UV deprotection of polypeptides and demonstrates the ability to selectively UV-deprotect polypeptides in the confined space of a nanoparticle dispersion.

## Introduction

Miniemulsion polymerization is an attractive technique for the synthesis of nanoparticles, where polymerization takes place in discrete surfactant-stabilized organic-phase nanodroplets in an aqueous medium (oil-in-water emulsion).^1−3^ While free radical polymerization is most frequently applied in miniemulsion polymerization, other polymerization techniques have been successfully explored.^4,5^ We have recently disclosed the first example of polypeptide nanoparticles obtained by miniemulsion, whereby the ring-opening polymerization of amino acid *N*-carboxyanhydrides (NCAs) was triggered in the oil phase stabilized by an amphiphilic glycosylated polypeptide surfactant.^6^ Further studies revealed a particle size dependence on the surfactant/core compatibility in that particles are reproducibly 20–30% larger if the hydrophobic surfactant block is identical to the amino acid polymerized in the core.^7^ We hypothesize that these polypeptide nanoparticles can have broad use particularly in pharmaceutical or personal care applications due to their innate biocompatibility and the fact that they are only comprised of natural amino acids and carbohydrate building blocks, which sets them apart from other nanoparticles typically obtained by miniemulsion polymerization.

To date, we have focused our investigation on hydrophobic amino acids such as leucine and phenylalanine for nanoparticles. To expand the range of nanoparticle properties, it would be desirable to introduce functional amino acids such as glutamic acid or lysine. However, by its nature, classical miniemulsion polymerization is restricted to hydrophobic, oil-phase soluble monomers.^8^ This can be overcome by applying inverse miniemulsion techniques by which hydrophilic functional nanoparticles, for example, from acrylic acid were obtained.^9−12^ In this setup, the polymerization occurs in water nanodroplets dispersed in an organic medium. However, this approach is not feasible for unprotected amino acid NCAs as their anhydride group is incompatible with the free carboxylic acid group of glutamic acid or the amino group of lysine and the aqueous medium. Here, we investigate the use of protected glutamate NCA and its selective deprotection post nanoparticle formation by conventional emulsion polymerization. Specifically, we aim to apply light for the selective deprotection inside the core of the nanoparticles. Light is a readily available stimulus, which creates a controlled level of reactivity based on its intensity and strength.^13,14^ Therefore, photocleavable moieties, such as *o*-nitrobenzyl (*o*-NB), coumarin, cinnamyl, and spiropyran groups, make a very attractive class of protecting groups for amino acids, which allow access to a range of cross-linking and deprotection chemistries.^15−21^ The use of light allows for selective deprotection within native conditions and has permitted for complex architectures to be synthesized.^22,23^ For the synthesis of polypeptide-based materials, *o*NB-lysine (*o*NB-Lys) and *o*NB-cysteine (*o*NB-Cys) are the most reported nitrobenzyl-functionalized amino acids. The Deming group was the first to report the synthesis of *o*NB-Lys, which was used to form photodegradable hydrogels.^22^ This was followed by reports by the Dong group where monomers were used to form hyperbranched polypeptides.^23^*o*NB-Lys was also polymerized using terminal amine poly(ethylene glycol) (PEG-NH_2_) as a macroinitiator and its subsequent deprotection and disassembly were monitored by UV–vis, Fourier transform infrared (FTIR), and dynamic light scattering (DLS).^24^ Alongside this work, Dong reported on the synthesis of *o*NB-Cys, which formed disulfide cross-links and after deprotection and oxidation produced a controlled drug release.^25^*o*NB-Cys has also been applied to synthesize other structures including hydrogels and nanoparticles by others.^26^ More recently, *o*NB-glutamate (*o*NB-Glu) has been synthesized and subsequently converted to NCA.^27^ This was polymerized using a PEG-NH_2_ initiator, which yielded a diblock copolymer with a controlled degree of polymerization and monomodal molecular weight distribution. The morphology achieved post UV irradiation was controlled through coordination of a metal center that was chelated to the exposed carboxylic acid moieties.

To better understand the photo deprotection of polypeptides, we conducted a systematic investigation into the effectiveness of light-mediated deprotection of *o*NB-Cys- and *o*NB-Glu-containing polypeptides. Initial studies were carried out on homopolypeptides in solution by varying the reaction parameters such as solvent, irradiation time, and molecular weight. Subsequently, the UV deprotection method was applied to (co)polypeptides within the confined environment of miniemulsion-templated nanoparticles.

## Experimental Section

### Materials

Unless otherwise noted, all reagents and chemicals were used as received without further purification. All amino acids, triphosgene, and 2-nitrobenzyl bromide were purchased from Fluorochem. Triethylamine, butylamine, and all solvents were purchased from Sigma-Aldrich, and HBr/acetic acid solution and allylamine were purchased from Alfa Aesar. All deuterated NMR solvents were purchased from Apollo Scientific Limited.

## Methods

*Nuclear magnetic resonance (NMR)* spectra were recorded on a Bruker Advance 400 MHz (^1^H). All chemical shifts (δ) are reported in parts per million (ppm) and analyzed relative to the residual nuclei of the reported deuterated solvent. CDCl_3_ (^1^H, δ = 7.26 ppm; ^13^C, δ = 77.16 ppm), *d*_6_-DMSO (^1^H, δ = 2.50 ppm; ^13^C, δ = 39.52), D_2_O (^1^H, δ = 4.79 ppm), and *d*-trifluoroacetic acid (^1^H, δ = 11.5 ppm; ^13^C, δ = 164.2 ppm). *Gel permeation chromatography (GPC)* was carried out using a PSS SECurity GPC system equipped with a PFG, 7 μm, 8 mm × 50 mm precolumn, a PSS 100 Å, 7 μm, 8 mm × 300 mm and a PSS 1000 Å, 7 μm, 8 mm × 300 mm column in series and a differential refractive index (dRI) detector at a flow rate of 1.0 mL min^–1^ in 1,1,1,3,3,3-hexafluoro-2-propanol (HFiP). *Dynamic light scattering (DLS)* analyses were carried out using a Malvern zetasizer nano ZSP instrument (Malvern Instruments, Malvern U.K.) with a detection angle of 173° and a 3 mW He–Ne laser operating at a wavelength of 633 nm. 1000 μL of solution was used to determine the size and distribution of the nanoparticles obtained. The measurements were carried out at 25 °C and a 10-fold dilution from the stock emulsion in disposable cuvettes. *Transmission electron microscopic (TEM)* images were recorded on a Hitachi H-7650 instrument at various magnifications. The samples (5 μL) were dropped on a Cu grid coated with SiO and Formvar and were wiped off after 10 min after which they were stained by a phosphotungstic acid stain (1% v/v in aqueous solution, 5 μL). Thermogravimetric analysis (TGA) was carried out on a TA TGA Q50 instrument under nitrogen flow using a platinum pan in the temperature range 30–600 °C.

### Homopolymers of *o*NB-Cys and *o*NB-Glu (General Procedure)

*o*NB-Cys or *o*NB-Glu NCA (200 mg) was dissolved in DMF (3 mL). Butylamine was added at the desired monomer to initiator ratio, after which visible bubbles appeared. The reaction was allowed to proceed for 48 h. The polypeptides were precipitated into diethyl ether (40 mL), collected by centrifugation, dissolved into chloroform (5 mL), and re-precipitated into diethyl ether (40 mL). The resulting solid was dried overnight in a vacuum oven to yield a white solid.

### General Miniemulsion Polymerization Procedure

A procedure previously described by Jacobs et al. was followed.^6^ The surfactant (80 mg) was dissolved in DDI water (10 mL), and the solution was allowed to cool in an ice bath for 10 min while stirring. The NCAs at desired ratios (70 mg) were dissolved in DCM (2 mL) and added to the aqueous solution dropwise while the reaction mixture was sonicated with a Hielscher ultrasonic processor UP200 St (*P* = 13 W, *c* = 100, *A* = 70%) for 15 min. Triethylamine (7 μL) was added and the system was allowed to stir (400 rpm) for 24 h at room temperature. The resulting particle dispersion was dialyzed (3.5 k *M*_W_ cutoff) against DI water for 3 days.

### Deprotection Procedure

**Scheme 1:**
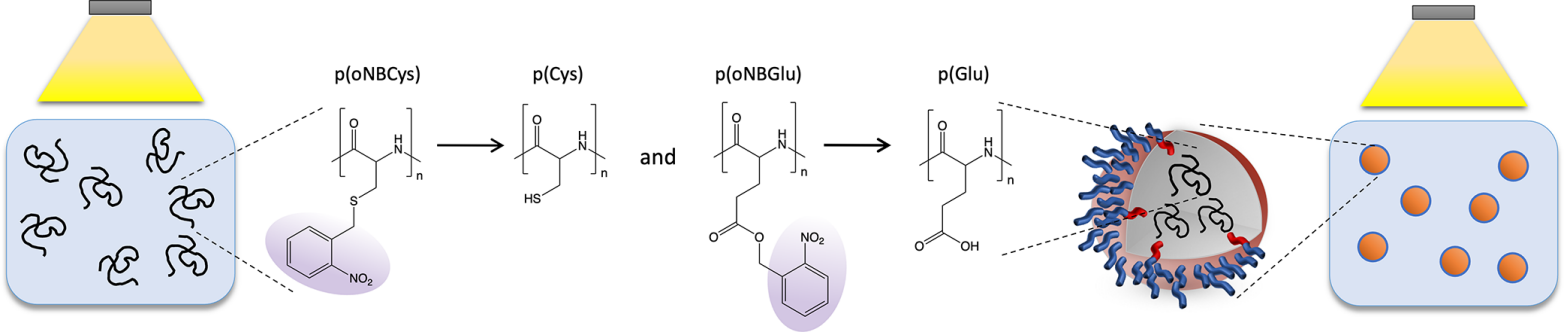
Light-Induced Deprotection of Poly(*o*-nitrobenzene-l-cysteine), p(*o*NB-Cys), and Poly(*o*-nitrobenzene-l-glutamate), p(*o*NB-Glu), in Solution and within the Core of Nanoparticles

All deprotection reactions were carried out using a Thorlabs UV-mounted 365 nm LED light source, M365L3 (*I* = 10.9 mW cm^–2^), and a 10 cm distance from the light source was maintained. NMR deprotection monitoring was carried out in NMR tubes, with the UV lamp angled at the solution from the side, and cleavage was determined by direct measurement in *d*_6_-DMSO solution. UV–vis deprotection of homopolypeptides was carried out using a 0.1 mg mL^–1^ solution in acetonitrile. 3 mL of the solution in a vial was exposed to the UV lamp directly above it. At each time point, 1 mL of the stock solution was removed for UV–vis measurement and then replaced to the stock for further exposure. Deprotection of miniemulsion nanoparticles was conducted in 25 mL vials (3 cm (D) × 5 cm (H)), which were lightly stirred during deprotection. The UV lamp was positioned directly above, and UV–vis measurements were recorded at 100-fold dilution in DI water (Scheme 1).

## Results and Discussion

**Figure 1:**
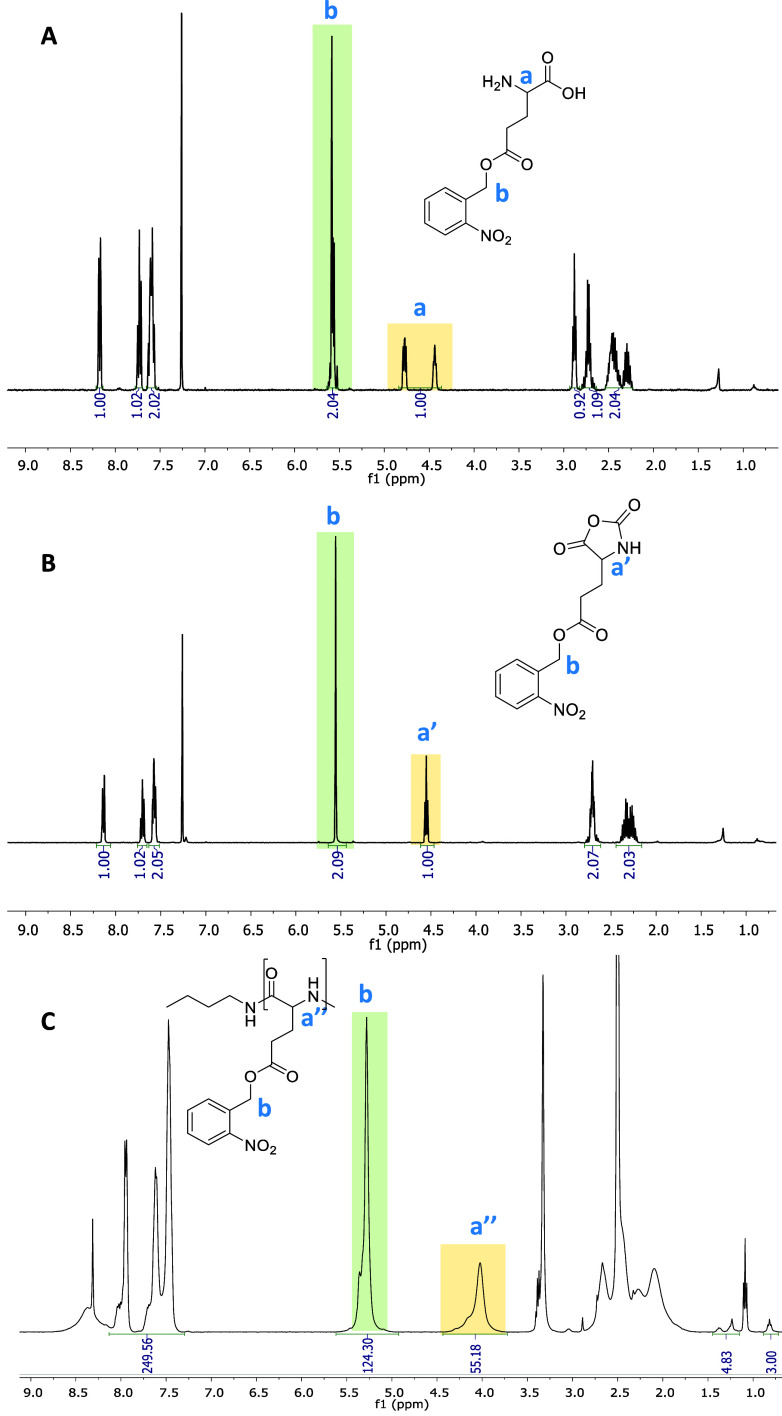
^1^H NMR spectra of the *o*-nitrobenzene l-glutamate (A), its NCA monomer (B), and poly(*o*-nitrobenzene-l-glutamate), p(*o*NB-Glu). Significant peaks are highlighted; for full assignment of spectra, see Figures S1–S17.

The synthesis of *o*NB-cysteine amino acid by the reaction of l-cysteine hydrochloride with *ortho*-nitrobenzyl bromide and its corresponding *N*-carboxyanhydride (NCA) is well reported, with high yields and facile methodology.^25^ Therefore, this was utilized here. However, the only report of the synthesis of *o*NB-glutamic acid uses harsh acidic conditions producing a moderate yield.^27^ Therefore, we explored a new route using the copper chelate method, which has been used in the past to synthesize other 5-glutamic acid esters under mild conditions.^28−30^ In the first step, a copper(II)–diglutamic acid complex is formed through chelation of the α-carboxylic acid groups (Scheme S2). This allowed for the direct and selective alkylation of the side-chain carboxylic acid group, which after decomplexation of copper, afforded the *o*NB-glutamate amino acid in a good yield (63% over the two steps). Both amino acids were converted to their respective NCA monomers with acceptable yields (69–75%) and purity and characterized extensively using NMR spectroscopy (Figures 1 and S1–S17).

No systematic study of the polymerization of these two NCAs and the UV deprotection of the corresponding polypeptides is available to date. Moreover, due to the confined environment within the droplet nanoreactors as well as the opaque nature of the miniemulsion, polymerization and UV deprotection monitoring were considered challenging. Therefore, studies were first carried out on homopolypeptides as models for the products formed in droplet nanoreactors. Preliminary solvent screening experiments confirmed that homopolymers (targeted degree of polymerization DP = 25) obtained in DMF produced monomodal size exclusion chromatography (SEC) traces with narrow dispersities (*Đ*) for both *o*NB-Glu and *o*NB-Cys. This was attributed to its ability to dissolve both the monomer and polypeptide unlike other solvents tested, such as THF and acetonitrile, which resulted in multimodal SEC traces and high dispersities (Figure S18). Next, the homopolymerization of both monomers was investigated targeting different DPs by varying the monomer to initiator ratio. The obtained DPs were calculated from ^1^H NMR spectra using end group analysis of −CH_3_ peak at 0.8 ppm (3H) of the butylamine initiator compared to the peak at 4.6 ppm of the backbone of the polypeptides (1H), Figure 1. For both polypeptides, good agreement was found between the targeted and calculated DPs (Table 1). However, GPC traces recorded in hexafluoro isopropanol (HFiP) suggested significantly less polymerization control for the p(*o*NB-Cys), exhibiting broad and monomodal traces particularly for lower DP samples (Figure S19). These results are broadly in line with those reported previously by Liu et al., who synthesized a p(*o*NB-Cys)_9_ homopolymer with a *Đ*_M_ = 1.45, although SEC was conducted with a DMF eluent which is known to report higher dispersities for polypeptides.^25^

**Table 1:** Poly(*o*-nitrobenzene-l-glutamate) and Poly(*o*-nitrobenzene-l-cysteine) Synthesized in DMF Targeting Different DPs (Initiator Butylamine)

polypeptide	DP_NMR_^a^	*M*_*n*NMR_ (g mol^–1^)^a^	*M*_*n*GPC_ (g mol^–1^)^b^	*Đ*_M_^b^
p(*o*NB-Glu)_10_	14	3700	10,300	1.2
p(*o*NB-Glu)_25_	22	5800	12,900	1.1
p(*o*NB-Glu)_35_	38	10,000	15,700	1.1
p(*o*NB-Glu)_50_	55	14,500	18,500	1.1
p(*o*NB-Glu)_100_	75	19,800	20,400	1.1
p(oNB-Cys)_10_	15	3600	7700	1.2
p(*o*NB-Cys)_25_	27	6400	9000	1.2
p(*o*NB-Cys)_50_	47	10,700	11,200	1.1
P(*o*NB-Cys)_100_	92	21,900	12,800	1.1

a Calculated by ^1^H NMR end-group analysis of terminal CH_3_ of butylamine initiator at 0.8 ppm compared to polypeptide backbone signal at 0.8 ppm (*d*_6_-DMSO, 400 MHz, 273 K).

b *M*_*n*_ and *Đ*_*M*_ values as calculated from PMMA standards using HFiP as the eluent.

**Figure 2:**
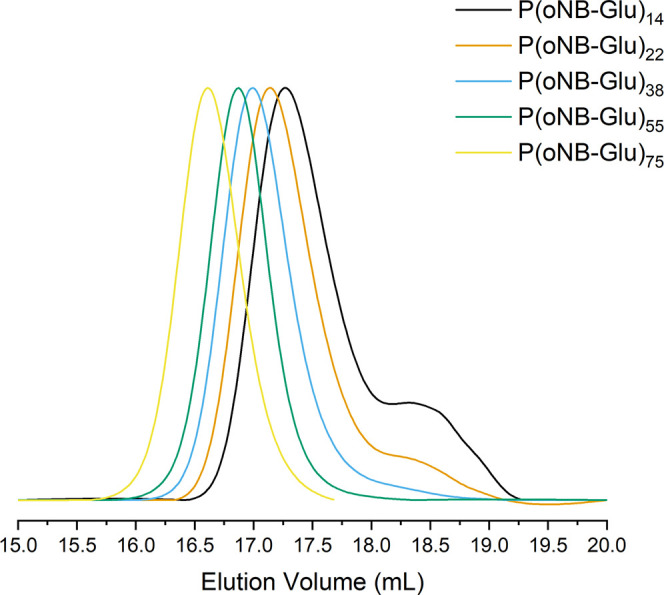
Size exclusion chromatography (SEC) traces of poly(*o*-nitrobenzene-l-glutamate), p(*o*NB-Glu), homopolypeptides with different targeted degrees of polymerization (DPs) using HFiP as the eluent. Molecular weights and dispersities (*Đ*) are listed in Table 1.

Following this, synthesis of p(*o*NB-Glu) of varying targeted DPs was attempted. The DP was calculated from ^1^H NMR spectroscopy using the −CH_3_ initiator peak at 0.8 ppm (3H) and the peak at 4.2 ppm of the backbone (1H), and the results confirm good agreement between the targeted and obtained DPs (Table 1). Moreover, the GPC traces of p(*o*NB-Glu) samples were much more symmetrical than those obtained for p(*o*NB-Cys) with a narrow dispersity, presenting clear shifts from low to high molecular weights (Figure 2). Low molecular weight fractions are usually ascribed to physically terminated β-sheet intermediates occurring at the early stage of the polymerization, which are more prominent for low DP samples as shown in Figure 2.^31^ There was no evidence of *o*NB cleavage during the polymerization as the product was a white solid. If unwanted cleavage had occurred in the polymerization, the product would be yellow; also, the cleavage product was absent in the NMR spectra obtained before UV irradiation.

Following the successful synthesis of the oNB-protected polypeptides, their UV-triggered cleavage was systematically studied. The cleavage at 365 nm of the *o*NB group is a well-reported phenomenon and as such its mechanism is well understood.^32−34^ The cleavage has been explored within many different architectures such as micelles, hydrogels, and so forth, while attached to different polymers such as poly(methacrylic acid) or as a linker between blocks.^26,35−38^ Photocleavage has mainly been tracked and proved using NMR and UV–vis spectrophotometry; however, it can also be qualitatively monitored as the nitrosobenzaldehyde side product turns the solution from clear to yellow color as it is produced.

^1^H NMR spectroscopy cleavage studies were first carried out in NMR tubes in DMSO-*d*_6_ as all products remain solvated throughout. The samples were irradiated for 2 h at 365 nm continuous wavelength at a 10 cm distance from the light source. Across the series of different DPs, the appearance of a singlet peak at 9.8 ppm after UV cleavage is indicative of the CH of the nitrosobenzaldehyde byproduct.^39^ Alongside the singlet, a doublet appears at ∼6.8 ppm in the p(Cys) spectra, which was assigned to the α-proton of the aldehyde, Figure S24. Moreover, the intensity of the peak at 4.05 ppm, representative of the CH_2_ of the *o*NB protecting group, reduces upon UV exposure. However, it does not completely disappear, indicating that full cleavage was not achieved for any of the p(*o*NB-Cys). The degree of cleavage was estimated to be around 8–13%, using integration of the aldehyde peak at 9.8 ppm. It must be noted that these calculations only provide indicative numbers due to the complexity of the reaction mixture and signal integration. The same study was carried out for the photo deprotection of p(*o*NB-Glu) to obtain p(Glu) using ^1^H NMR spectroscopy in *d*_6_-DMSO. Once again, the formation of benzaldehyde is clear as for all molecular weights, the tell-tale peak at 9.8 ppm is present, Figure S25. Alongside this, a broad peak at ∼12 ppm appears which was assigned as the carboxylic acid proton. Comparably to the p(Cys) series, the benzylic protons of the *o*NB group (5.3 ppm) decreased in intensity after UV exposure but once again did not entirely disappear. For this polymer, the degree of cleavage was calculated by utilizing the peak at 12 ppm, affording 2, 18, 27, 31, and 34% for P(Glu)_14_, P(Glu)_22_, P(Glu)_38_, P(Glu)_55_, and P(Glu)_75_, respectively. Therefore, it can be concluded that by NMR spectroscopy, the cleavage at 365 nm after 2 h did not reach full conversion, which could be a result of the ability of nitrosobenzaldehyde to act as an internal light filter at concentrations required for NMR spectroscopy on polymeric species.^40,41^

**Figure 3:**
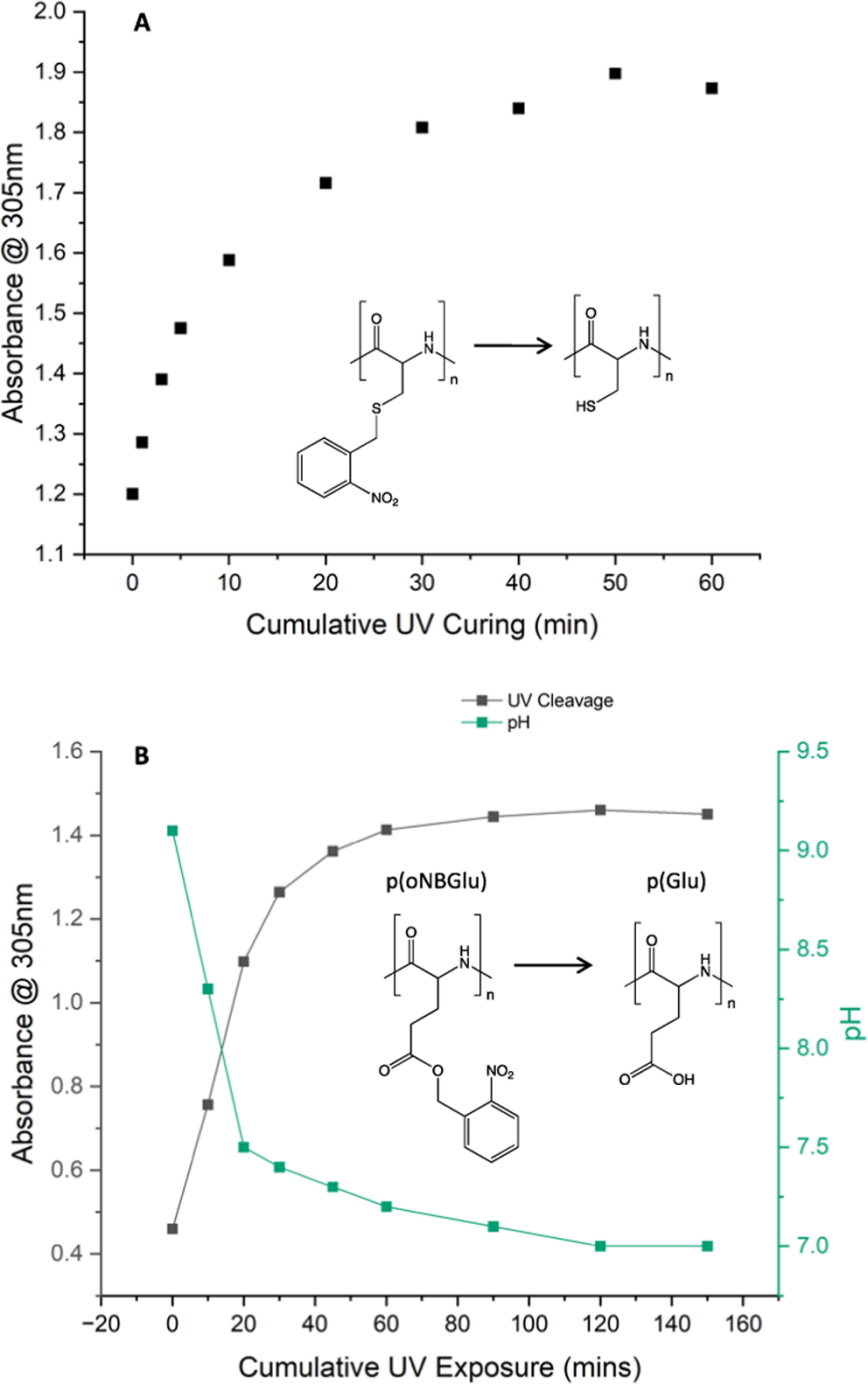
Cleavage of the *o*NB group from poly(*o*-nitrobenzene-l-cysteine)_27_ (A) and poly(*o*-nitrobenzene-l-glutamate)_22_ (B) upon irradiation at 365 nm in acetonitrile. Cumulative UV–vis absorbance at 305 nm plotted against time and pH change.

Therefore, following NMR studies, 0.1 mg mL^–1^ solutions in acetonitrile of the homopolypeptides were exposed to 365 nm light, and the UV–vis spectra were recorded at given time points. Acetonitrile was chosen as the solvent because its UV absorbance cutoff is 190 nm compared to DMSO at 265 nm, and it was able to solubilize the polypeptides at the required concentrations. According to Liu et al., the cleavage of *o*NB from p(*o*NB-Cys)-containing PEG blocks occurred rapidly over the first 20 min and then leveled off.^25^ The rate of cleavage is heavily variable, controlled by the power of the light source, distance from it, and so forth; so, using the previous reports as a guideline, the cleavage was tracked over the first 60 min for homopolypeptides of p(*o*NB-Cys), Figure S26. Contrary to NMR spectroscopy, UV–vis spectroscopy monitors the production of nitrobenzaldehyde without using the resulting polypeptide for reference. As seen from the traces, the cumulative cleavage increases as the peak at 305 nm increases in intensity, which is characteristic for *o*NB.^42^ To enable a more observable visualization of the cleavage, the absorbance at 305 nm was plotted against cumulative UV exposure, Figures 3A and S27. All curves leveled out around 40 min for all molecular weights, indicating the absence of a molecular weight-dependent *o*NB removal. These results qualitatively agree with the NMR studies, suggesting a low deprotection rate. For the p(*o*NB-Glu) series, however, it was found that during the first 60 min, the absorbance peak at 305 nm did not reach a plateau for all polypeptides, Figure S28. Therefore, the cleavage was investigated over a longer period of 360 min with fewer time points taken in the early stages to minimize the volume change caused by extensive sampling. Alongside the absorbance, pH was also measured. As can be seen in Figures 3B and S29, the traces produced by the prolonged exposure are far more uniform in shape compared to those in Figure S28, which alludes to the fact that a longer exposure time is required. The peak absorbance at 305 nm reaches a plateau between 75 and 100 min, except for that of the lowest molecular weight, p(*o*NB-Glu)_14_, which concludes around 50 min. This therefore indicates that for p(*o*NB-Glu) molecular weight does impact the rate of cleavage, which we hypothesize may be caused by the increase in hydrophilicity as the degree of deprotection increases, potentially affecting chain arrangement and hydrodynamic volume. This was mirrored in the pH decreasing as the free carboxylic acids of p(Glu) are revealed after photo deprotection. The starting solutions of p(*o*NB-Glu) measured pH 9 because acetonitrile is a strong hydrogen bond acceptor and as such can be considered weakly basic compared to the aqueous solutions used to calibrate the pH meter. It is expected that this will influence the pH of the solution measured and as such the decrease in pH seen may not be as drastic as expected; however, the trend accurately mirrored the UV–vis spectra and so was considered qualitatively accurate. Considering the p(*o*NB-Glu) series, the degree of cleavage follows the same trend of molecular weight dependence by NMR spectroscopy and UV–vis spectroscopy.

**Figure 4:**
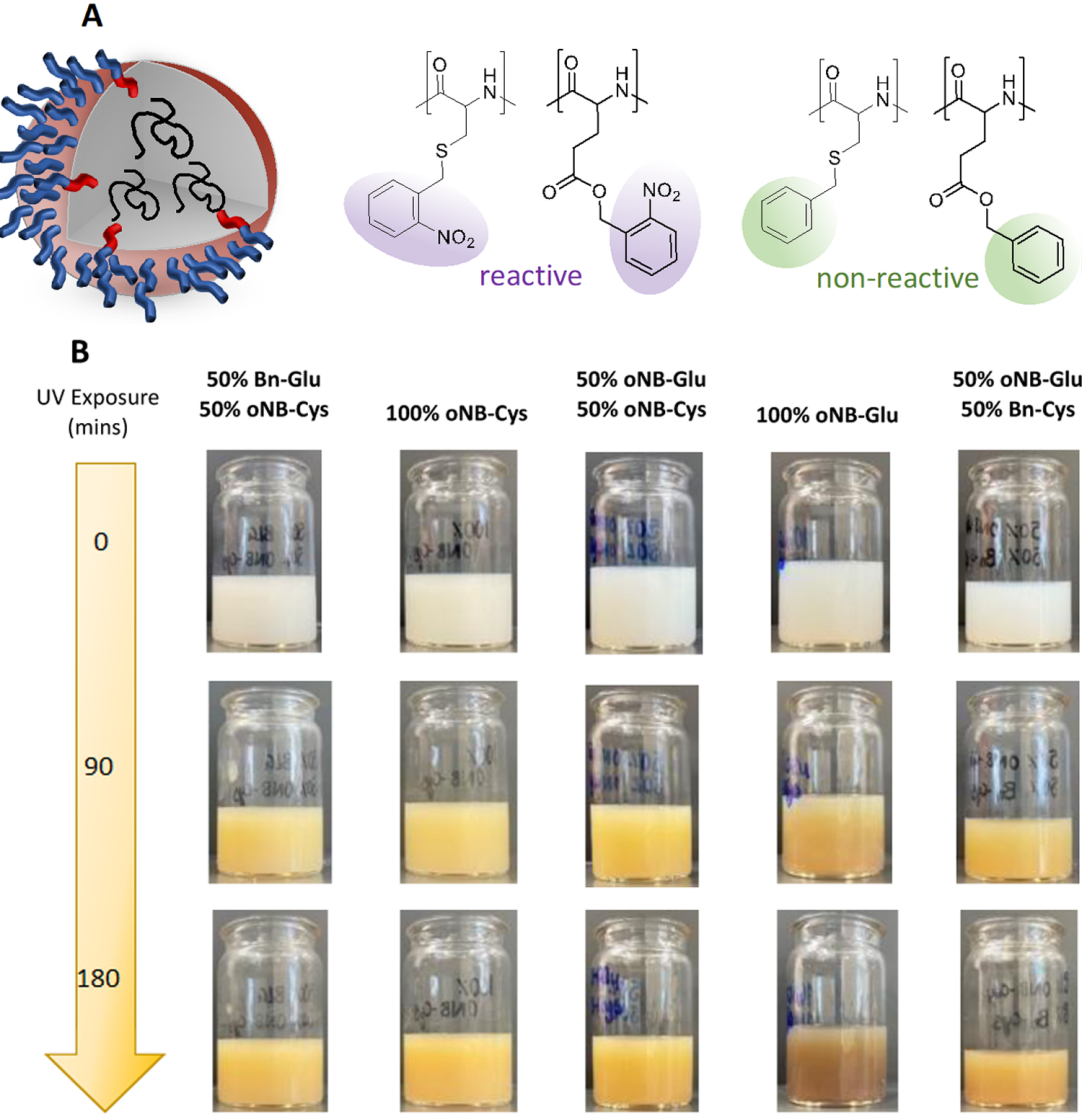
(A) Graphical representation of the composition of the nanoparticles composed of polypeptides with photoreactive protecting groups (purple) and those without (green). (B) Images of the nanoparticle miniemulsion series over the course of UV irradiation (365 nm) exhibiting qualitatively the cleavage as the solutions turn yellow.

Building on the results from the homopolypeptides, next, it was investigated whether the deprotection process can be transferred to polypeptide particles from miniemulsion. Previous work in our group has elucidated the synthesis of polypeptide nanoparticles obtained through NCA ring-opening polymerization (ROP) via miniemulsion.^6,7^ In the initial work, *o*NB-Cys was used as the sole monomer, and it was hypothesized that upon cleavage of the *o*NB group, the exposed free thiols would be forming disulfide bonds creating cross-links. Here, we systematically investigated *o*NB-Cys and *o*NB-Glu copolymers at different monomer ratios as core forming polypeptides. Benzyl-l-glutamate (Bn-Glu) and benzyl-l-cysteine (Bn-Cys) were used as additional nonreactive monomers, Figure 4A and Table 2. It was envisaged that *o*NB-Glu, once cleaved, will reveal a free pendant carboxylic acid, which will result in anionic charge and increased hydrophilicity of the core of the emulsion-templated nanoparticles.

**Table 2:** *Z*-Average Diameters as Recorded by DLS for Miniemulsion Polymerization

monomers		mass ratio (%)		dialyzed^a^		UV exposed^b^		dialysis post UV exposure^c^	
glu variant	cys variant	glu	cys	size (nm)	PDI	size (nm)	PDI	size (nm)	PDI
*n*-Glu		100		109	0.19				
Bn-Glu	*o*NB-Cys	75	25	129	0.19	129	0.19	126	0.18
Bn-Glu	*o*NB-Cys	50	50	137	0.13	139	0.14	137	0.14
Bn-Glu	*o*NB-Cys	25	75	142	0.10	137	0.11	142	0.11
	*o*NB-Cys		100	157	0.10	154	0.12	157	0.10
*o*NB-Glu	*o*NB-Cys	25	75	142	0.10	141	0.10	141	0.11
*o*NB-Glu	*o*NB-Cys	50	50	128	0.11	130	0.12	129	0.12
*o*NB-Glu	*o*NB-Cys	75	25	124	0.11	123	0.11	123	0.12
*o*NB-Glu		100		125	0.16	121	0.17	115	0.15
*o*NB-Glu	Bn-Cys	75	25	135	0.10	124	0.11	117	0.12
*o*NB-Glu	Bn-Cys	50	50	140	0.10	134	0.12	132	0.14
*o*NB-Glu	Bn-Cys	25	75	163	0.21	160	0.21	161	0.21
	Bn-Cys		100	172	0.22				

a 72 h purification by dialysis.

b UV exposed for ca. 360 min.

c 72 h purification by dialysis after UV cleavage.

The nanoparticles were synthesized using our previously reported o/w emulsion polymerization methodology, using a macromolecular glycopolypeptide surfactant containing lysine functionalized with lactobionic acid, Scheme S4.^6,7^ The aqueous phase consisted of 80 mg of surfactant dissolved in 10 mL of water and a DCM oil phase of 2 mL containing a total mass of 70 mg of monomers at different compositions. Triethylamine initiator was added after sonication and the suspension was polymerized open to air for 24 h, after which the particles were purified by dialysis for 72 h. During the synthesis and purification, the particles were kept out of ambient light to allow for a controlled tracking of the UV cleavage. Figure S31 indicates that cleavage did not occur during synthesis as the nanoparticle solution remained as a white turbid solution. The nanoparticle’s sizes were measured by DLS after 24 h polymerization followed by dialysis, after UV exposure, and again after dialysis post UV exposure to investigate any differences in the hydrodynamic diameter. No significant *Z*-average size changes were detected for any of the investigated particles between these processing steps (Table 2 and Figures S32–S37). All nanoparticles were below 200 nm, most of them below 150 nm with narrow PDI values and smooth correlograms, indicating the absence of aggregates. All traces seen for both intensity and number-average are monomodal throughout without the presence of any free surfactant.

**Figure 5:**
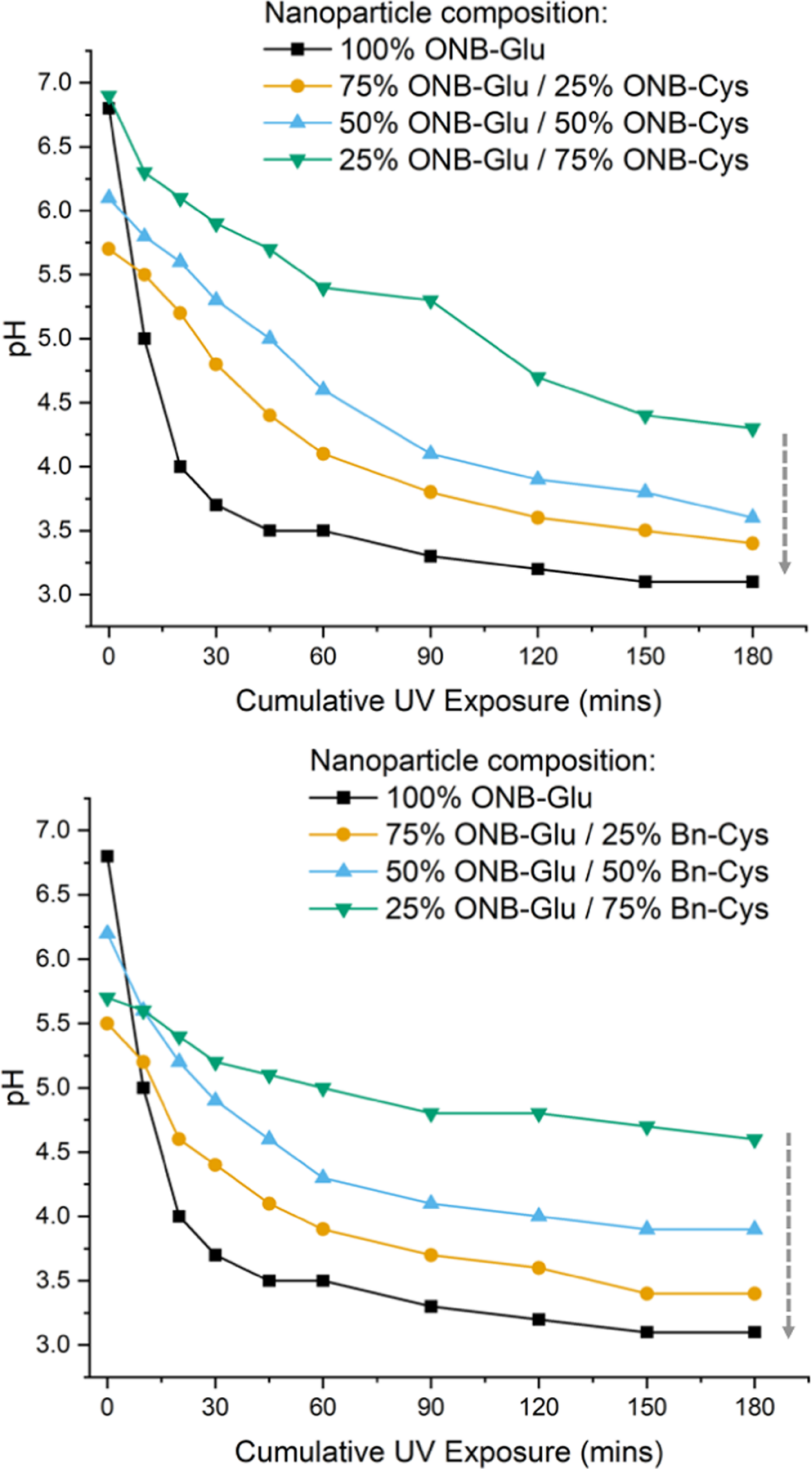
pH changes associated with nanoparticles containing p(*o*NB-Glu-*co*-*o*NB-Cys) (A) and p(*o*NB-Glu-*co*-Bn-Cys) copolypeptides upon UV irradiation (365 nm) of the miniemulsion post NCA polymerization. The arrow indicates increase in *o*NB-monomers in the polypeptides.

Using the results obtained for homopolypeptides, the emulsions were exposed to UV light under a 365 nm continuous wavelength light source. However, due to the turbidity of the milky white nanoparticle suspension, the penetration of light was envisaged to be inefficient, and as such it was expected that UV deprotection would be less efficient than that for the polypeptides in solution. Visual inspection of the UV-irradiated samples, however, showed a clear yellowing of the samples for all investigated monomer compositions. The discoloration increases with increasing irradiation time for all samples, Figures 4B and S38–S48. Notably, solutions that initially contained more *o*NB-Glu became brown after 180 min UV exposure compared to *o*NB-Cys which remained yellow, in agreement with the observed different deprotection rates for the respective polypeptides in solution. Further evidence for the successful core deprotection was obtained from the fact that the aqueous solution of nanoparticles containing p(*o*NB-Glu) did produce an apparent change to lower pH upon irradiation (Figure 5), which is consistent with the results obtained for the polypeptides in solution (Figure 3). Alongside this, the most obvious pH change was seen when 100% *o*NB-Glu was present. Initially, the solution measured pH 6.7; however, after 180 min the solution measured pH 3.1, in agreement with the higher concentration of pendant carboxylic acid groups within the core of the nanoparticle. This provides clear evidence for the successful deprotection in the confined core environment, which appears to follow the same reactivity as observed for the respective polypeptides in solution. While the results provide qualitative evidence for the successful deprotection inside the nanoparticles, none of the samples exhibited the indicative UV–vis peak at 305 nm for nitrosobenzaldehyde in the aqueous medium, Figures S38–S48. Moreover, attempts to remove nitrosobenzaldehyde from the nanoparticles were unsuccessful. Even after extensive dialysis, the nanoparticles retained the yellow color, which implies that nitrosobenzaldehyde remains trapped in the core of the nanoparticle. We hypothesize that this could be caused by hydrophobic interactions and π–π stacking with the poly(l-Phe) block of the surfactant which forms an effective diffusion barrier at the nanoparticle/water interface. Therefore, further studies into the effect of different (nonaromatic) hydrophobic surfactant blocks on the diffusion of small molecules need to be carried out.

## Conclusions

Utilizing *o*-nitrobenzyl-protected cysteine and glutamate NCAs, we synthesized a set of copolymers in solution and in nanodroplets of an o/w miniemulsion and investigated the UV deprotection to reveal the native amino acid pendant groups. UV deprotection in solution could be monitored by ^1^H NMR and UV–vis spectroscopy as well as pH monitoring (for the *o*NB-Glu) and revealed a molecular weight dependence of the deprotection efficiency for the *o*NB-Glu series, which was not seen for the *o*NB-Cys. Following this, the monomers were successfully incorporated into a miniemulsion polymerization which enabled the synthesis of core functionalized nanoparticles. While UV deprotection within the core of nanoparticles was envisaged to be more challenging due to the solution turbidity and the confined core space, color change from white to yellow/brown and a drop of the solution pH for the p(*o*NB-Glu) samples indicate successful deprotection. Moreover, the degree of color and pH change logically followed the concentration of cleavable *o*NB-Glu and *o*NB-Cys in the nanoparticles. However, it was found that the deprotection byproduct, the yellow nitrosobenzaldehyde, could not be removed from the nanoparticles. This has implications for the use of these polypeptide nanoparticles as small molecule drug delivery materials, possibly preventing diffusion-based release. It needs to be further investigated to what extent this phenomenon is dependent on the structure of the small molecule, the nature of the amino acids forming the core, and the type of nanoparticle surfactant.

## Supporting Information Available

Experimental procedures; additional NMR and IR spectra of polymers; DLS data and TEM images of nanoparticles; DLS data of shell-modified nanoparticles; additional tables with reactant ratios and particle sizing (PDF)
