# Supplementary material for: Investigation of the Effectiveness of Photo Deprotection of Polypeptides in Solution and within the Core of Miniemulsion-Derived Nanoparticles
Source: Macromolecules. 2024 Feb 27;57(5):1979–87. doi: 10.1021/acs.macromol.3c02538 (PMC10938878; doi:10.1021/acs.macromol.3c02538)
Supplement: Supplementary file 1 — ma3c02538_si_001.pdf [file ma3c02538_si_001.pdf]

Investigation of the Effectiveness of Photodeprotection of Polypeptides in Solution and Within the Core of Miniemulsion Derived Nanoparticles

Nicola Judge[1] and Andreas Heise*[1,2,3]

[1]Department of Chemistry, RCSI University of Medicine and Health Sciences, Dublin, D02 YN77, Ireland

[2]Science Foundation Ireland (SFI) Centre for Research in Medical Devices (CURAM), RCSI, Dublin, D02 YN77, Ireland

[3]AMBER, The SFI Advanced Materials and Bioengineering Research Centre, RCSI, Dublin, D02 YN77, Ireland.

1.0. Experimental Methods

N-Carboxyanhride (NCA) monomers of Z-L-Lysine, L-Phe, Bn-L-Glutamate and Bn-L-Cysteine were synthesised according to literature procedures.

1.1. *ortho*-Nitrobenzyl Cysteine

Ortho-Nitrobenzyl Cysteine was synthesised according to previous published procedures. L-Cysteine hydrochloride (5 g, 31.72 mmol) and *ortho*-Nitrobenzyl bromide (6.23 g, 28.8 mmol) were dissolved in suspended in acetonitrile (150mL). NaOH (2.31g, 57.8 mmol) was dissolved in DI water (30mL) and added dropwise and the resulting solution allowed to stir for 2hrs. The precipitate was collected and washed with a 1:1 water/acetonitrile. The product was recrystalised from acetone: IPA: H2O (5:5:20mL per 1g crude) affording a white solid (83% yield).

^{1}H NMR (400 MHz, $CDCl_3$/CF_3OOD (5:1)): δ 8.09 (1H, d, J = 8.1 Hz), 7.74 – 7.60 (m, 1H), 7.58 – 7.42 (m, 2H), 4.93 (1H, m), 4.22 (m, 2H), 3.19 (1H, m), 3.11 (1H, m). ^{13}C (75 MHz, $CDCl_3$/CF_3OOD (5:1)): δ 174.81, 163.27, 162.84, 162.40, 161.97, 148.39, 134.61, 133.36, 132.59, 129.64, 126.45, 118.85, 116.03, 113.21, 110.39, 77.48, 77.16, 76.84, 52.90, 34.70, 33.01.

1.2. *ortho*-Nitrobenzyl Cysteine NCA

Ortho-nitrobenzyl cysteine NCA was synthesised according to a modified procedure. [1,5] *Ortho*-nitrobenzyl cysteine (5g, 19.55 mmol) was suspended in THF (100mL), epichlorohydrin (7.23g, 78.11 mmol) was added and the solution heated to 50°C. Triphosgene (2.89g, 9.76 mmol) was dissolved in THF (20mL) and added dropwise the reaction was allowed to proceed until all solids had dissolved. The solution was concentrated in vacuo to 1/3rd of the volume and precipitated into hexane. The solid was collected and recrystallised twice (THF/hexane) before being dried under vacuum to afford a white solid (75%).

^{1}H NMR (400 MHz, $CDCl_3$/ *d*-TFA (5:1)): δ 8.10 (1H, d, J= 8.3 Hz), 7.65 (1H, m), 7.51 (2H, m), 4.67 (1H, m), 4.16 (2H, m), 3.14 (1H, dd, J= 14.7, 3.7 Hz), 2.95 (1H, dd, J= 14.7, 6.2 Hz); ^{13}C (75 MHz, $CDCl_3$/ *d*-TFA (5:1)): δ 168.12, 155.55, 148.42, 134.33, 133.07, 132.51, 129.43, 126.10, 58.42, 34.50, 33.06.

1.3. Copper glutamic acid complex, Cu(Glu)$_2$

L-Glutamic acid copper (II) complex was synthesized according to previously published procedure. Copper (II) acetate monohydrate (20.6 g, 103 mmol) in DI water (375 mL) was added dropwise to a solution of L-glutamic acid (14.7 g, 100 mmol) in DI water (375 mL) at 70˚C. The mixture was cooled and allowed to crystallize over 2 days. The precipitate was collected and washed with water, ethanol and ether and dried in the vacuum oven (40 ˚C, 24hrs). The product was isolated as a blue powder (85%).

TGA: 130-200 ˚C – 4 H_2O; 250-500 ˚C, decomposition; >550 ˚C, residue 2CuO.

1.4. *ortho*-Nitrobenzyl glutamate

Ortho-nitrobenzyl glutamate was synthesized according to a modified procedure. Cu(L-Glutamic acid)$_2$ (4.10 g, 6.57 mmol) and L-Glutamic Acid (1.93 g, 13.14 mmol) was suspended in DMF (20mL) and DI water (5mL) which was dissolved upon dropwise addition of N,N,N',N'-tetramethytlguanidine (4.4 mL, 35mmol). The reaction was heated to 40 ˚C, after all solids dissolved (90 minutes), additional DMF (25mL) was added. *Ortho*-Nitrobenzyl bromide (5.68 g, 26.3 mmol) was added in one portion, after 2 hrs extra DMF (50 mL) was added and over the following 36 hrs extra DMF was added (100 mL) to prevent solidification. The reaction was precipitated into excess acetone and stirred until a fine powder was obtained, the solid was collected by vacuum filtration and dried in-*vacuo*. The product was stirred in a saturated EDTA (100 mL) solution for 24 hrs, the product was collected as the decomplexation repeated until the filtrate was no longer blue. The product was further purified by recrystallization from acetone: IPA: H_2O (5:5:20 mL per 1g of crude) affording a white solid (74% yield).

^{1}H NMR (400 MHz, $CDCl_3$/ *d*-TFA (5:1)): δ 8.17 (1H), 7.72 (1H), 7.60 (2H), 5.57 (2H), 4.44 (1H), 2.75 (2H), 2.40 (2H). ^{13}C (75 MHz, $CDCl_3$/D-TFA (5:1)): δ 175.8, 175.6, 147.8, 135.1, 130.8, 130.7, 130.4, 126.1, 65.7, 52.9, 30.7, 26.2.

1.5. ortho-Nitrobenzyl glutamate NCA

Ortho-Nitrobenzyl glutamate NCA was synthesized according to a modified procedure. Ortho-Nitrobenzyl glutamate (2.475 g, 8.77 mmol) was suspended in THF (150 mL), epichlorohydrin (3.24 g, 35.08 mmol) was added and the solution heated to 50 ˚C. Triphosgene (2.89 g, 9.76 mmol) was added in one portion and the reaction was allowed to proceed until all solids had dissolved. The solution was concentrated in vacuo to 1/3rd of the volume and precipitated

into hexane. The solid was collected and recrystallized twice (THF/hexane) before being dried under vacuum to afford a white solid (69%).

^{1}H NMR (400 MHz, $CDCl_3$/ *d*-TFA (5:1)): δ 8.14 (1H), 7.70 (1H), 7.59 (2H), 5.56 (1H), 4.55 (1H), 2.70 (2H), 2.34 (2H); ^{13}C (75 MHz, $CDCl_3$/D-TFA (5:1)): δ 173.5, 168.6, 158.3, 147.8, 134.3, 130.5, 130.3, 130.0, 125.6, 64.9, 56.9, 29.5, 26.6.

1.6. Homopolypeptide of photocleavable monomers

P(oNB-Cys)$_{10}$ homopolypeptide: *Ortho*-Nitrobenzyl Cysteine NCA (200 mg, 0.708 mmol) was dissolved in DMF (3mL). Butylamine (7 µL, 0.071 mmol) was added in one portion, visible bubbles appeared, and the reaction was allowed to proceed for 48 hrs. The reaction was precipitated into diethyl ether (40mL), collected by centrifugation, dissolved into chloroform (5mL) and reprecipitated into diethyl ether (40mL). The resulting solid was dried overnight in the vacuum oven to give a white solid (89%).

P(oNB-Cys)$_{25}$ homopolypeptide: Previously described procedure, with modified quantities of *ortho*-Nitrobenzyl Cysteine NCA (200 mg, 0.708 mmol) was dissolved in DMF (3mL), butylamine (2.8 µL, 0.028 mmol) was added.

P(oNB-Cys)$_{50}$ homopolypeptide: Previously described procedure, with modified quantities of *Ortho*-Nitrobenzyl Cysteine NCA (200 mg, 0.708 mmol) was dissolved in DMF (3mL), butylamine (1.4 µL, 0.014 mmol) was added.

P(oNB-Cys)$_{100}$ homopolypeptide: Previously described procedure, with modified quantities of *Ortho*-Nitrobenzyl Cysteine NCA (200 mg, 0.708 mmol) was dissolved in DMF (3mL), butylamine (0.7 µL, 0.007 mmol) was added.

P(oNB-Glu)$_{10}$ homopolypeptide: Previously described procedure, with modified quantities of *Ortho*-Nitrobenzyl Glutamate NCA (200 mg, 0.76 mmol) was dissolved in DMF (3mL), butylamine (7.5 µL, 0.076 mmol) was added.

P(oNB-Glu)$_{25}$ homopolypeptide: Previously described procedure, with modified quantities of *Ortho*-Nitrobenzyl Glutamate NCA (200 mg, 0.76 mmol) was dissolved in DMF (3mL), butylamine (3 µL, 0.03 mmol) was added.

P(oNB-Glu)$_{35}$ homopolypeptide: Previously described procedure, with modified quantities of *Ortho*-Nitrobenzyl Glutamate NCA (200 mg, 0.76 mmol) was dissolved in DMF (3mL), butylamine (2.1 µL, 0.02 mmol) was added.

P(oNB-Glu)$_{50}$ homopolypeptide: Previously described procedure, with modified quantities of *Ortho*-Nitrobenzyl Glutamate NCA (200 mg, 0.76 mmol) was dissolved in DMF (3mL), butylamine (1.5 µL, 0.015 mmol) was added.

P(oNB-Glu)$_{100}$ homopolypeptide: Previously described procedure, with modified quantities of *Ortho*-Nitrobenzyl Glutamate NCA (200 mg, 0.76 mmol) was dissolved in DMF (3mL), butylamine (0.75 µL, 0.007 mmol) was added.

1.7. Glycopolypeptide Surfactant Synthesis

Synthesized according to literature procedure[1]

Scheme S1: Mini emulsion polymerisation glycopolypeptide surfactant synthesis. NCA Z-L-Lys (1) dissolved in DMF, 0°C and initiator allyamine added to afford P(Z-L-Lys) (2) L-Phe

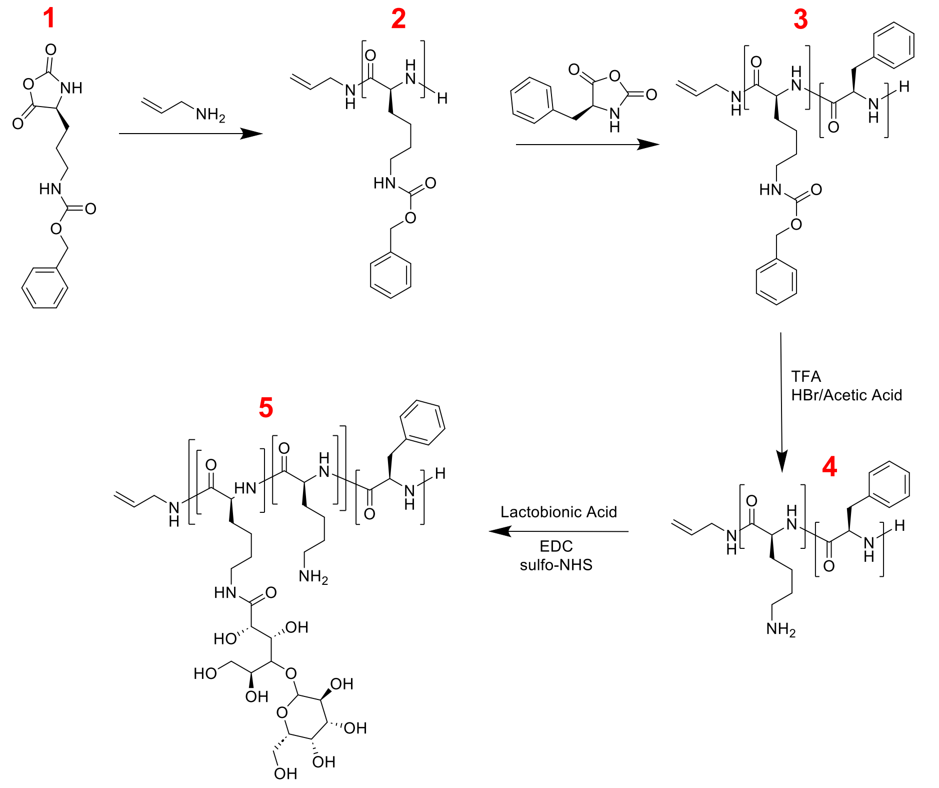

Block-co-polypeptide Synthesis

Z-L-Lysine NCA (Z-L-Lys NCA) (9 g, 29.38 mmol) was dissolved in DMF (55 mL) and placed under vacuum at 0 °C. Allylamine (44 µL, 0.58 mmol) was dissolved in DMF (1 mL) and added in one injection. The reaction proceeded for 5 days under vacuum at 0 °C. L-

---

[1] Jacobs, J.; Pavlović, D.; Prydderch, H.; Moradi, M.-A.; Ibarboure, E.; Heuts, J. P. A.; Lecommandoux, S.; Heise, A. Polypeptide Nanoparticles Obtained from Emulsion Polymerization of Amino Acid N-Carboxyanhydrides. *J. Am. Chem. Soc.* **2019**, *141*, 12522.

Phenylalanine NCA (L-Phe NCA) (1.12g, 5.88 mmol) was dissolved in DMF (4 mL) and added to the solution, the reaction proceeded overnight. The reaction was precipitated into diethyl ether (3 x 500 mL) and then dried under vacuum overnight.

^{1}H NMR (400 MHz, *d*-TFA, δ) 7.31 (m), 5.71 (s), 5.19 (d), 4.77 (s), 4.58 (d), 3.21 (s), 2.96 (s), 2.06 – 1.15 (m). SEC $Đ_M$ - 1.15, M_w -16,800 g mol^{-1}.

Z-L-Lysine Deprotection

The polypeptide (4 g, 0.32 mmol with respect to lysine repeat units) was dissolved in TFA (16 mL). HBr solution (33% in acetic acid, 3-fold excess with respect to Z-L-Lys units) was added to the solution dropwise whilst stirring in an ice bath. The reaction was left to proceed overnight and then precipitated into diethyl ether (2 x 250 mL) and then dialysed (3.5k Mw cut off) against DDI water for 3 days before being lyophilised.

^{1}H NMR (400 MHz, D_2O) δ 4.20 (m), 2.89 (m), 1.83 – 1.44 (m), 1.44 – 1.13 (m). SEC $Đ_M$ - 1.05, M_w -14,500 g mol^{-1}.

Glycosylation

Lactobionic acid (1.15 g, 3.23 mmol, 15-fold excess with respect to lysine repeat units), 1-Ethyl-3-[3- dimethylaminopropyl]-carbodiimide hydrochloride (EDC) (618.2 mg, 3.23 mmol) and N-hydroxysulfosuccinimide (Sulfo-NHS) (70 mg, 0.32 mmol) were dissolved in MES buffer (7.2 mL, 10 mM, pH 4.7) and stirred for 20 minutes. This was then added to a solution of polypeptide (1.8 g, 0.22 mmol) in DDI water (14 mL). The reaction was stirred overnight and then purified by dialysis (3.5k Mw cut off) for 3 days and lyophilised.

^{1}H NMR (400 MHz, D_2O) δ 4.54 (d), 4.29 (m), 4.15 (s), 4.08 (s), 3.99 – 3.64 (m), 3.56-3.52 (m), 2.99 (m), 1.70 – 1.43 (m). SEC $Đ_M$ - 1.06, M_w -15,100 g mol^{-1}.

1.8. General miniemulsion polymerisation procedure.

As previously described by Jacobs et al. The surfactant (80 mg), was dissolved in DDI water (10 mL) and solution cooled in an ice bath for 10 minutes whilst stirring. NCA (70 mg) was dissolved in DCM (2 mL) and added to the aqueous solution dropwise while the reaction mixture was sonicated with Hielscher Ultrasonic Processor UP200 St (P= 13 W, c= 100, A= 70 %) for 15 min. Triethylamine (7 µL) was added and the system allowed to stir (400 rpm) for 24hs at room temperature. The resulting particle dispersion was dialysed (3.5k Mw cut off) against DDI for 3 days.

Table 1. Photoreactive nanoparticle emulsion formulations

Monomer(s)		% by mass		Surfactant (mg)	Glu Monomer (mg)	Cys Monomer (mg)	Initiator (µL)
Glu Variant	Cys Variant	% Glu	% Cys				
Bn-Glu	-	100		80	70	-	7
Bn-Glu	oNB-Cys	75	25	80	52.5	17.5	7
Bn-Glu	oNB-Cys	50	50	80	35	35	7
Bn-Glu	oNB-Cys	25	75	80	17.5	52.5	7
	oNB-Cys		100	80	-	70	7
oNB-Glu	oNB-Cys	25	75	80	17.5	52.5	7
oNB-Glu	oNB-Cys	50	50	80	35	35	7
oNB-Glu	oNB-Cys	75	25	80	52.5	17.5	7
oNB-Glu	-	100		80	70	-	7
oNB-Glu	Bn-Cys	75	25	80	52.5	17.5	7
oNB-Glu	Bn-Cys	50	50	80	35	35	7
oNB-Glu	Bn-Cys	25	75	80	17.5	52.5	7
	Bn-Cys		100	80	-	70	7

4.0. Additional Figures

Scheme S2: Methodology for the synthesis of oNB-Glu NCA beginning with the formation of $Cu(Glu)_2$ complex (a) to which oNB-bromide is then coupled to the ε-carboxylic acid (b). The functionalised amino acid is revealed by chelating the Cu(II) ions with EDTA (c) which is then cyclised in the presence of triphosgene to give the photoreactive oNB-Glu NCA (d).

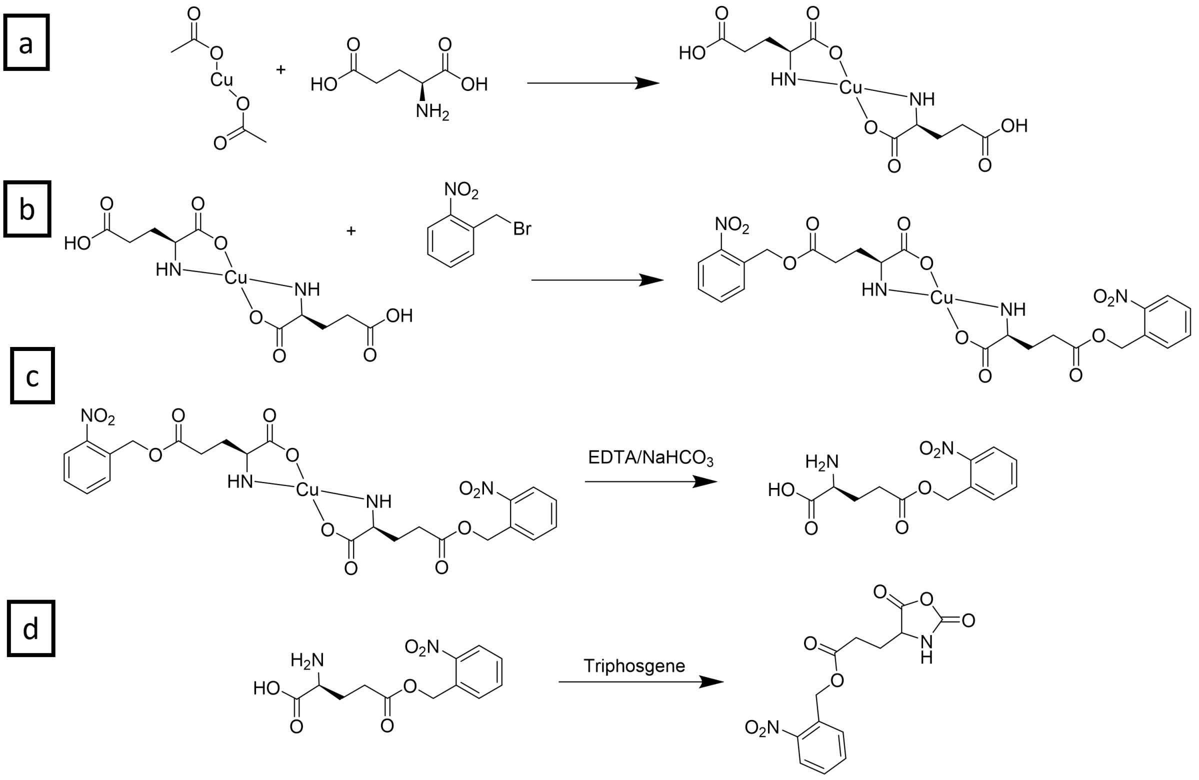

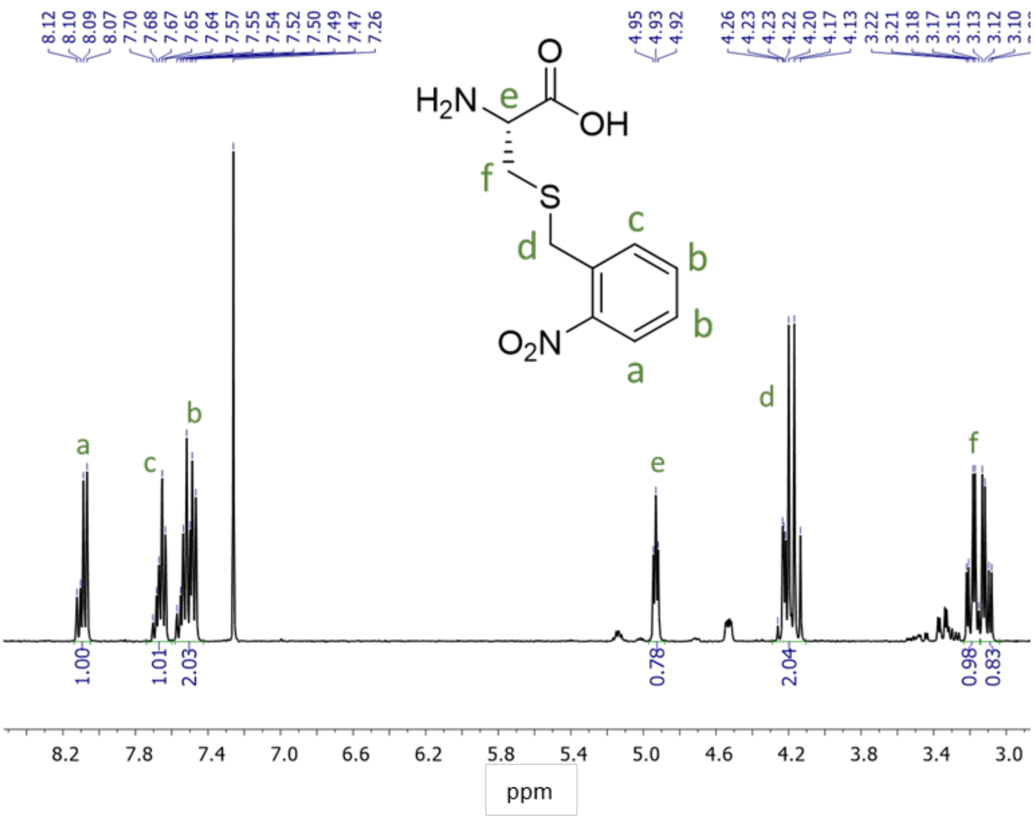

Figure S1: ^{1}H NMR spectra ($CDCl_3$/d-TFA (1:1)) of oNB-Cysteine functionalised amino acid including assignments.

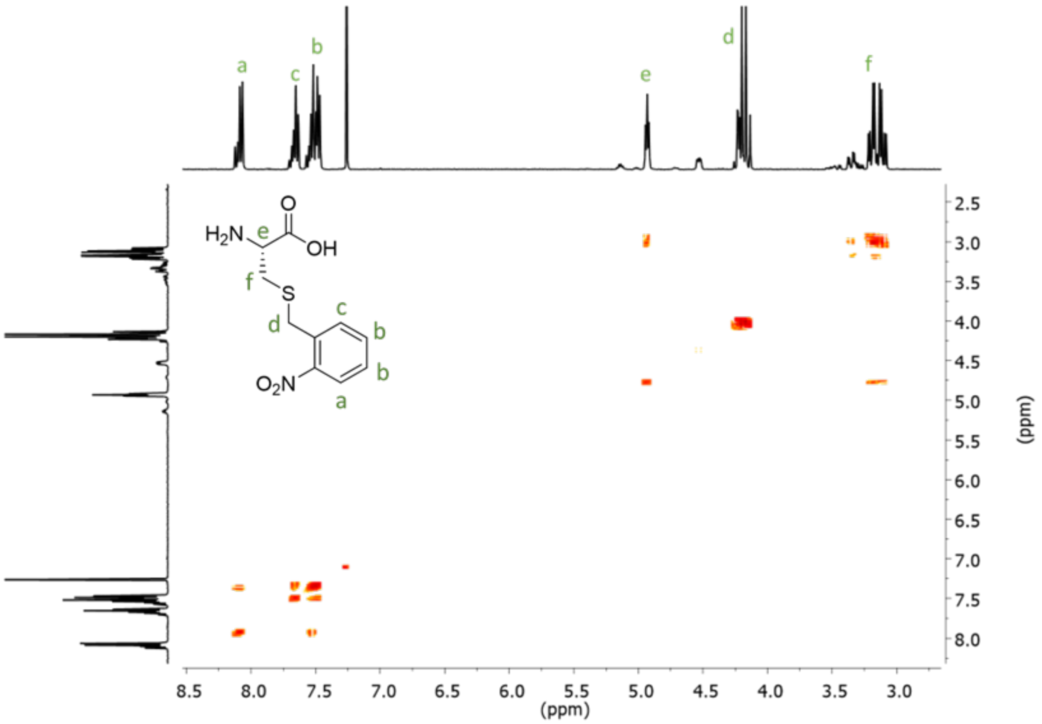

Figure S2: ^{1}H COSY NMR spectra ($CDCl_3$/d-TFA (1:1)) of oNB-Cysteine functionalised amino acid.

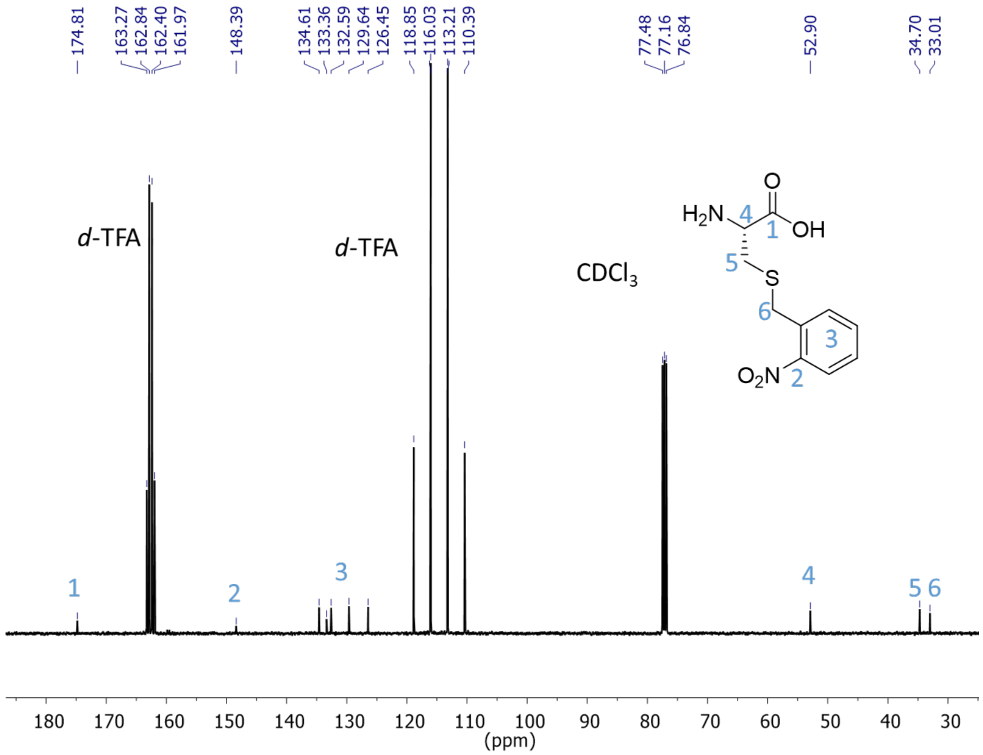

Figure S3: ^{13}C NMR spectra ($CDCl_3$/d-TFA (1:1)) of oNB-Cysteine functionalised amino acid including assignments.

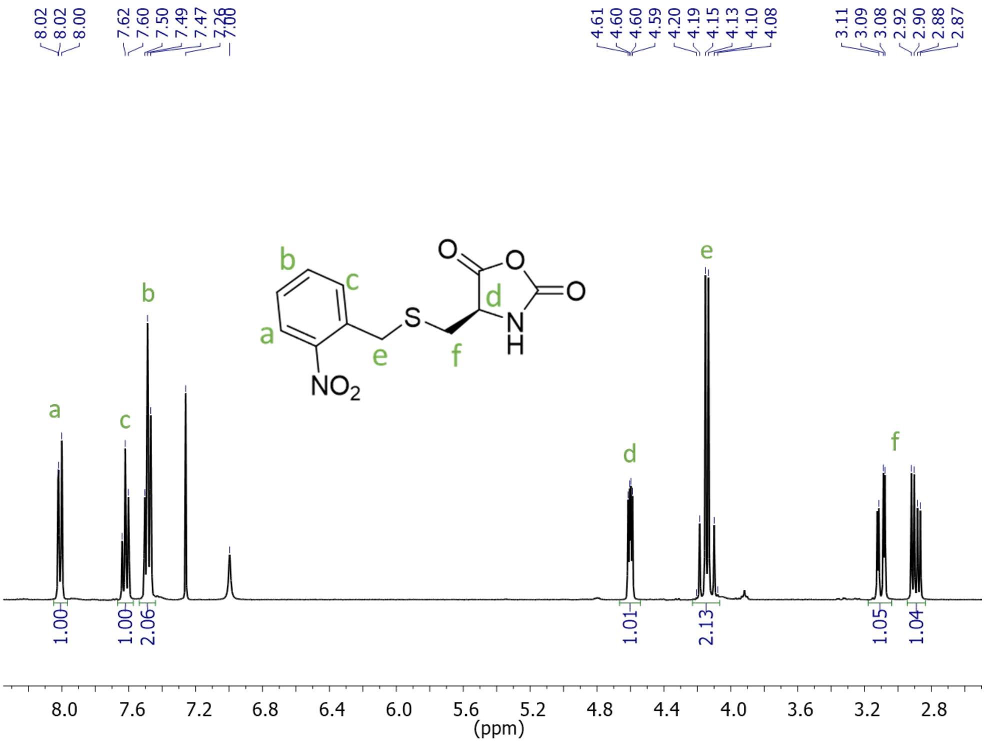

Figure S4: ^{1}H NMR spectra ($CDCl_3$/d-TFA (1:1)) of oNB-Cysteine NCA including assignments.

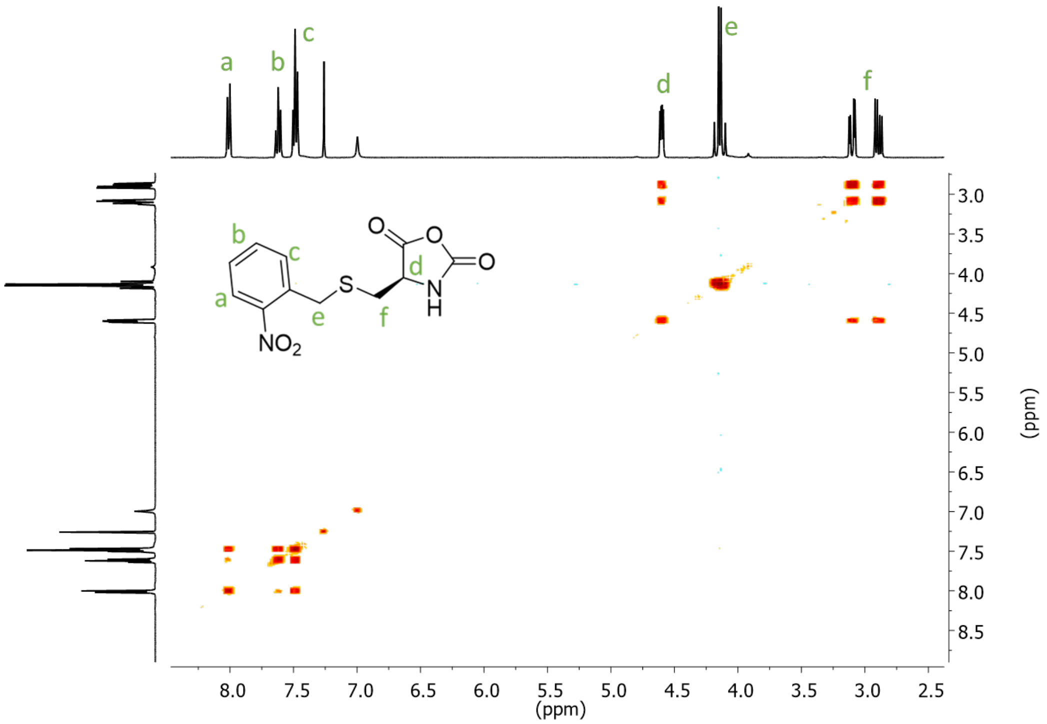

Figure S5: ^{1}H COSY NMR spectra ($CDCl_3$/d-TFA (1:1)) of oNB-Cysteine NCA.

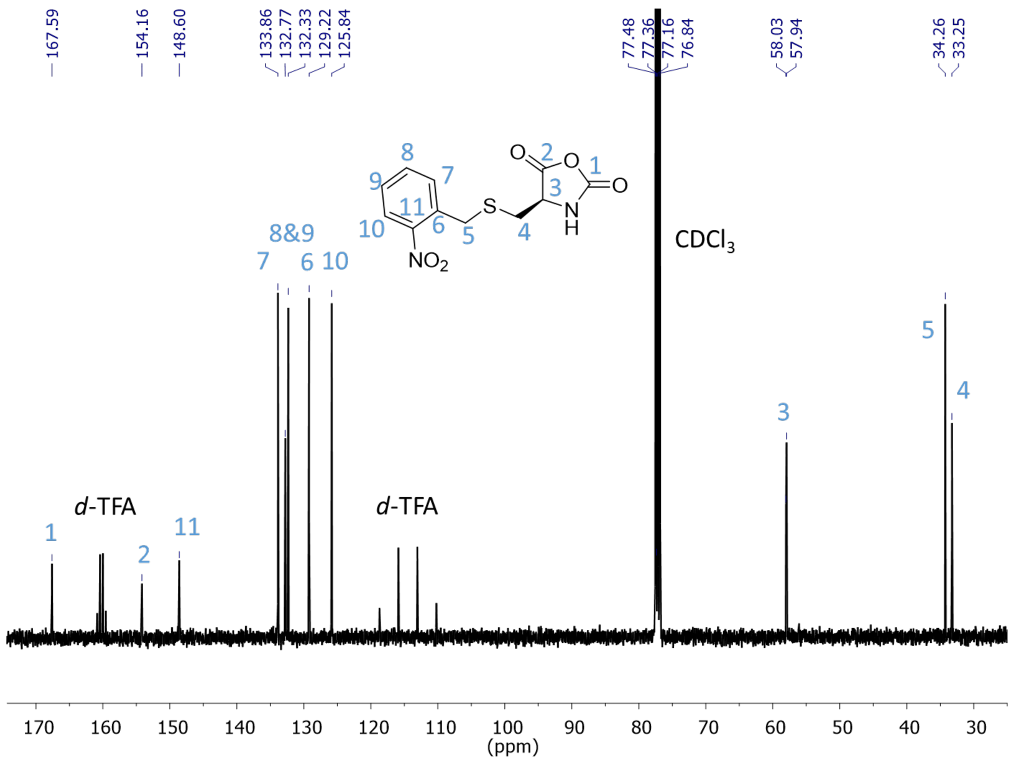

Figure S6: ^{13}C NMR spectra ($CDCl_3$/d-TFA (1:1)) of oNB-Cysteine NCA including assignments.

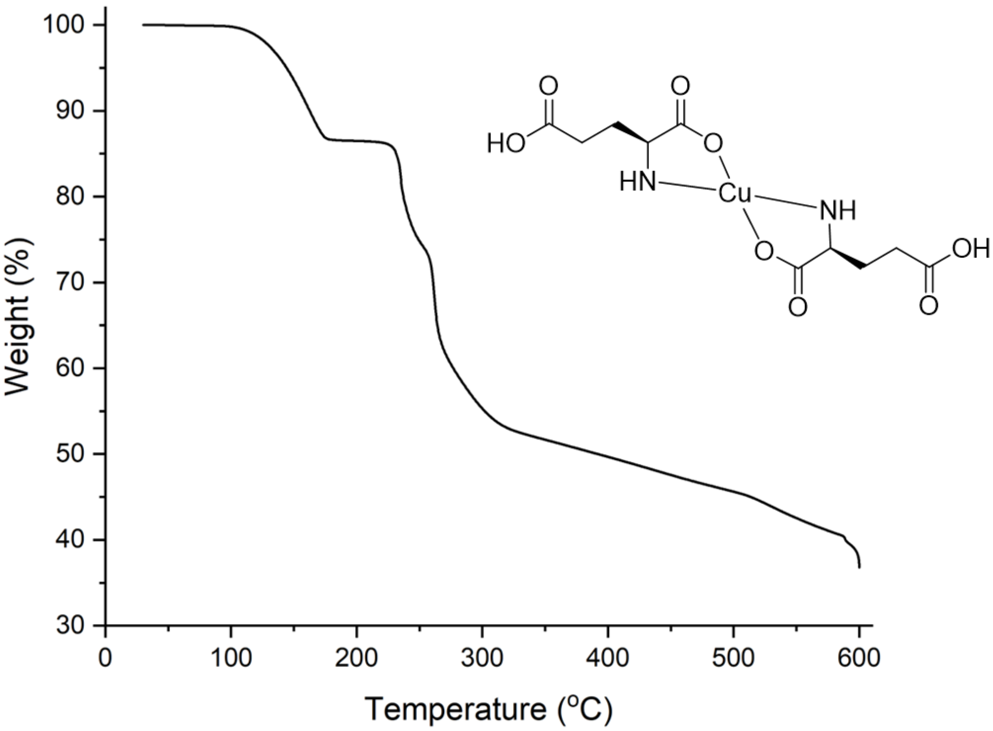

Figure S7: Thermogravimetric Analysis trace of $Cu(Glu)_2$

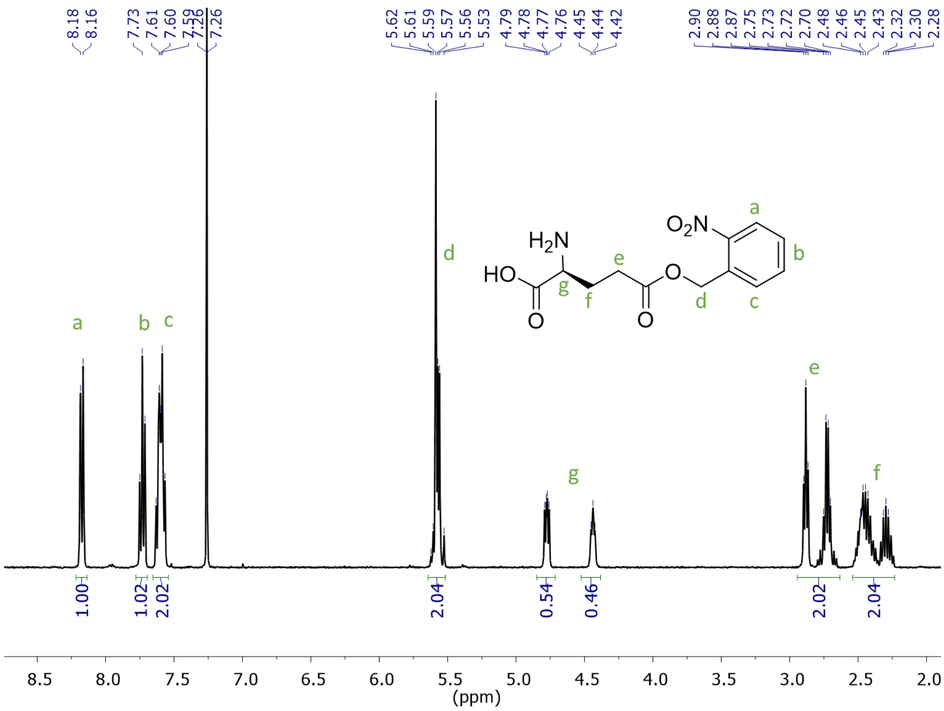

Figure S8: ^{1}H NMR spectra ($CDCl_3$/d-TFA (1:1)) of oNB-Glutamate amino acid including assignments.

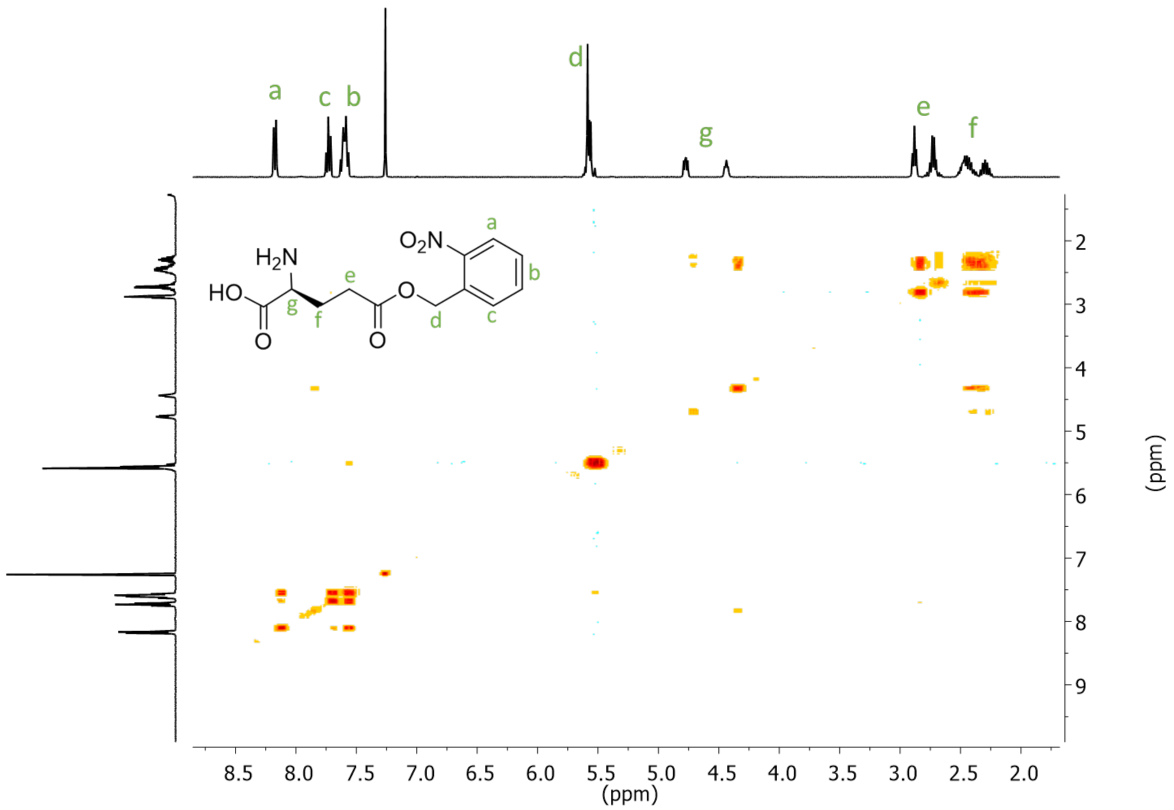

Figure S9: ^{1}H-^{1}H COSY NMR spectra ($CDCl_3$/d-TFA (1:1)) of oNB-Glutamate amino acid including assignments.

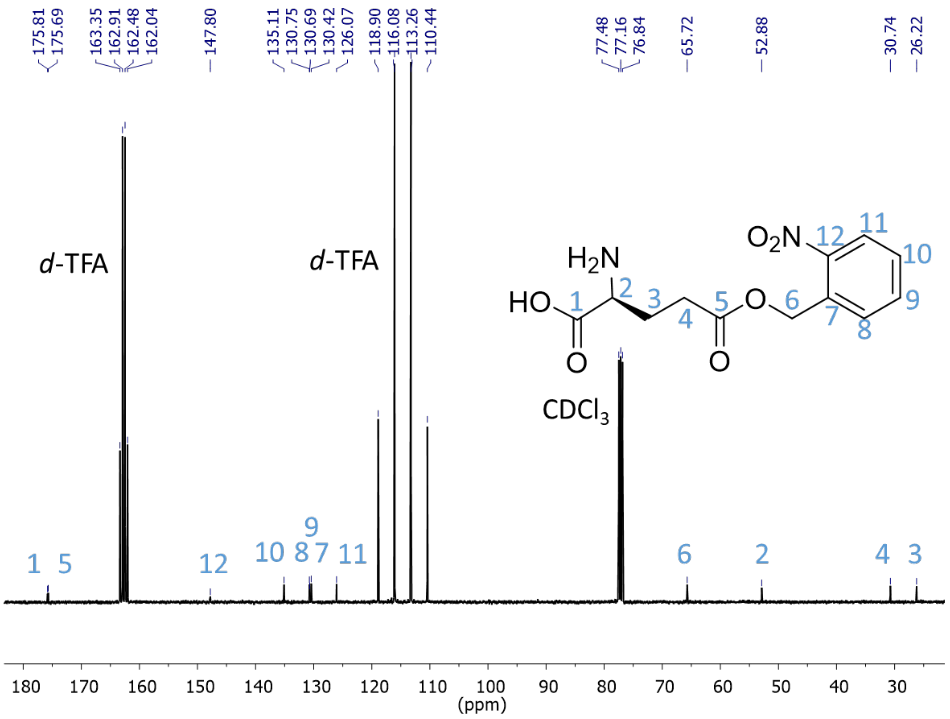

Figure S10: ^{13}C NMR spectra ($CDCl_3$/d-TFA (1:1)) of oNB-Glutamate amino acid including assignments.

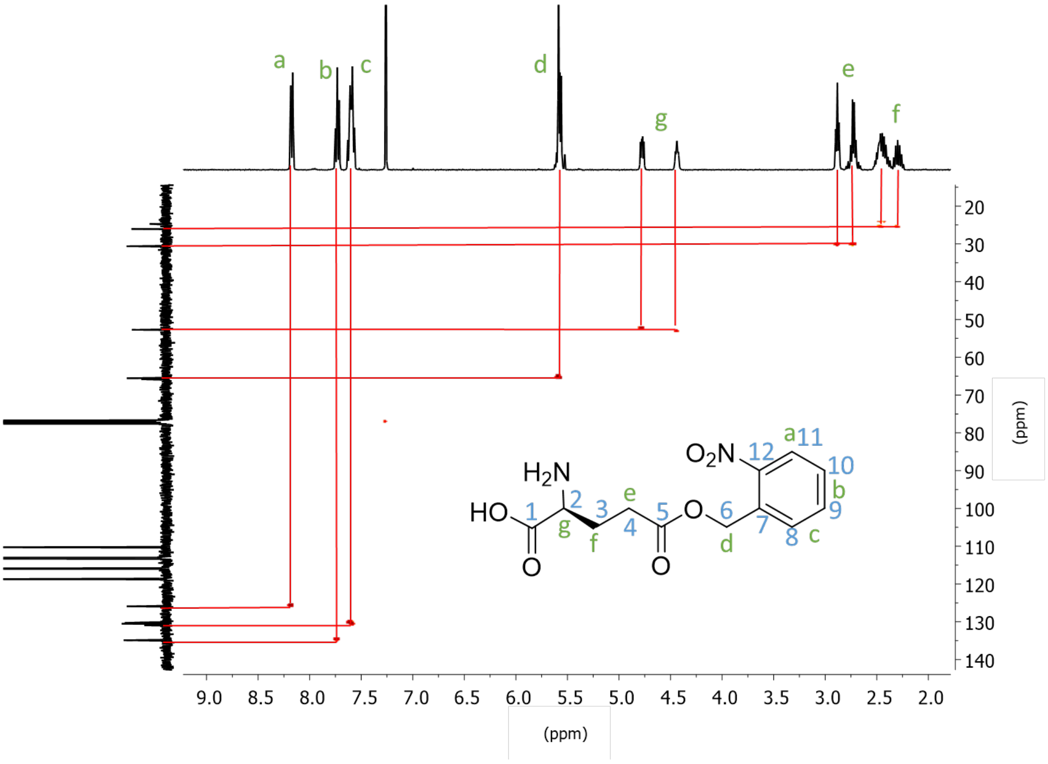

Figure S11: ^{1}H-^{13}C HSQC NMR spectra ($CDCl_3$/d-TFA (1:1)) of oNB-Glutamate amino acid including assignments.

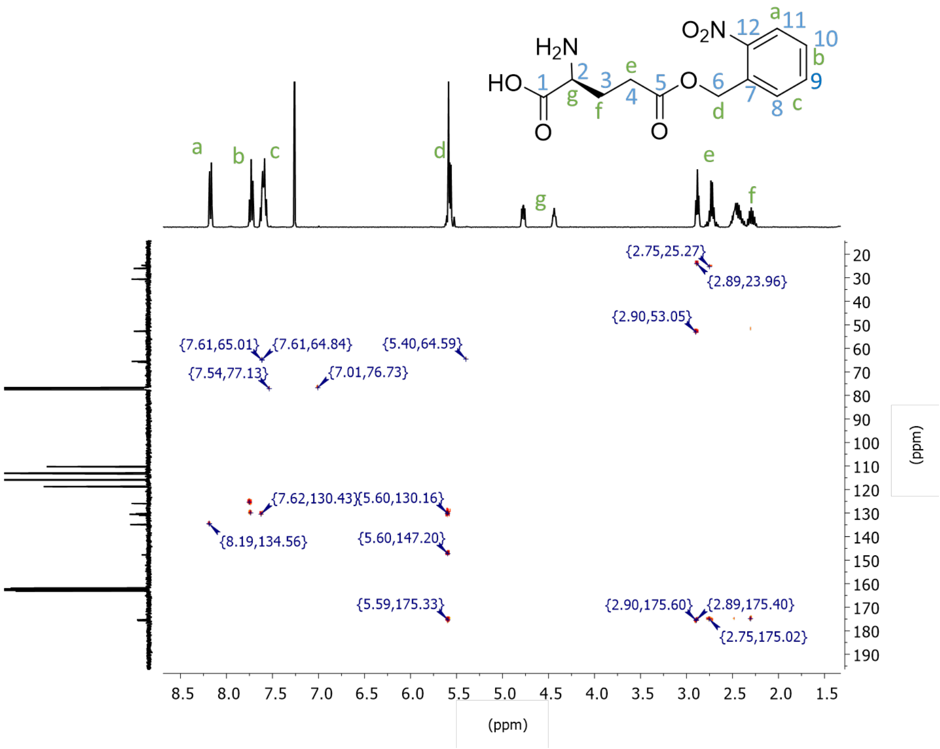

Figure S12: ^{1}H-^{13}C HMBC NMR spectra ($CDCl_3$/d-TFA (1:1)) of oNB-Glutamate amino acid including assignments.

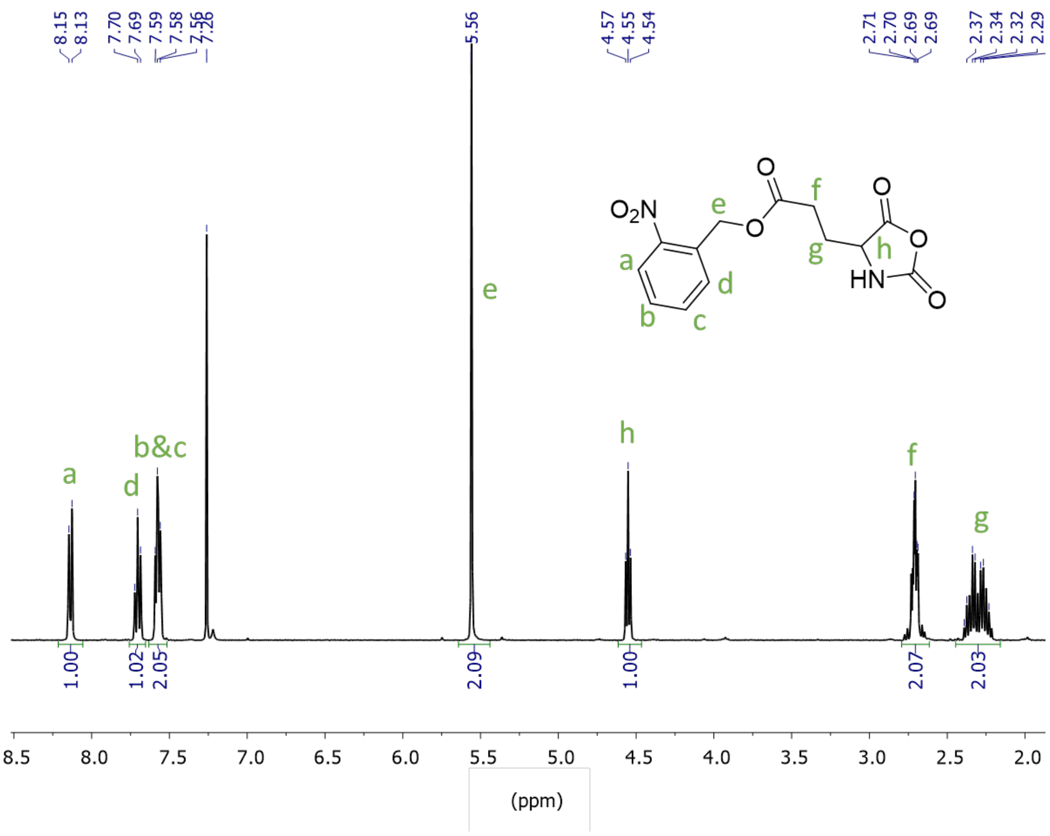

Figure S13: ^{1}H NMR spectra (CDCl$_3$/d-TFA (1:1)) of oNB-Glutamate NCA including assignments.

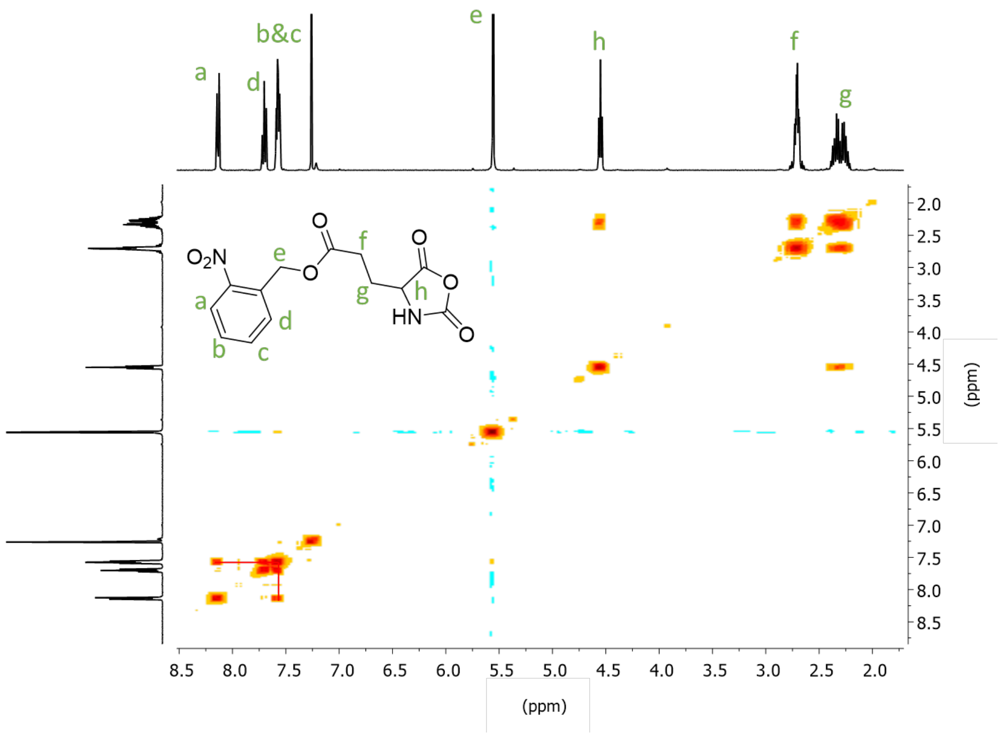

Figure S14: ^{1}H-^{1}H COSY NMR spectra (CDCl$_3$/d-TFA (1:1)) of oNB-Glutamate NCA including assignments.

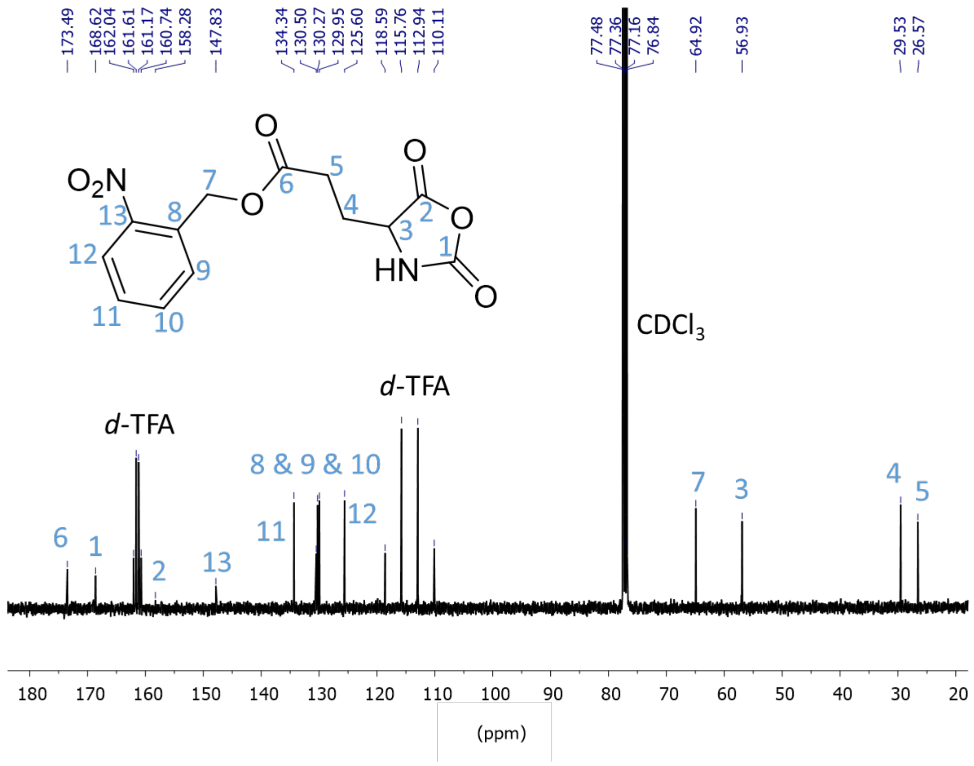

Figure S15: ^{13}C NMR spectra ($CDCl_3$/d-TFA (1:1)) of oNB-Glutamate NCA including assignments.

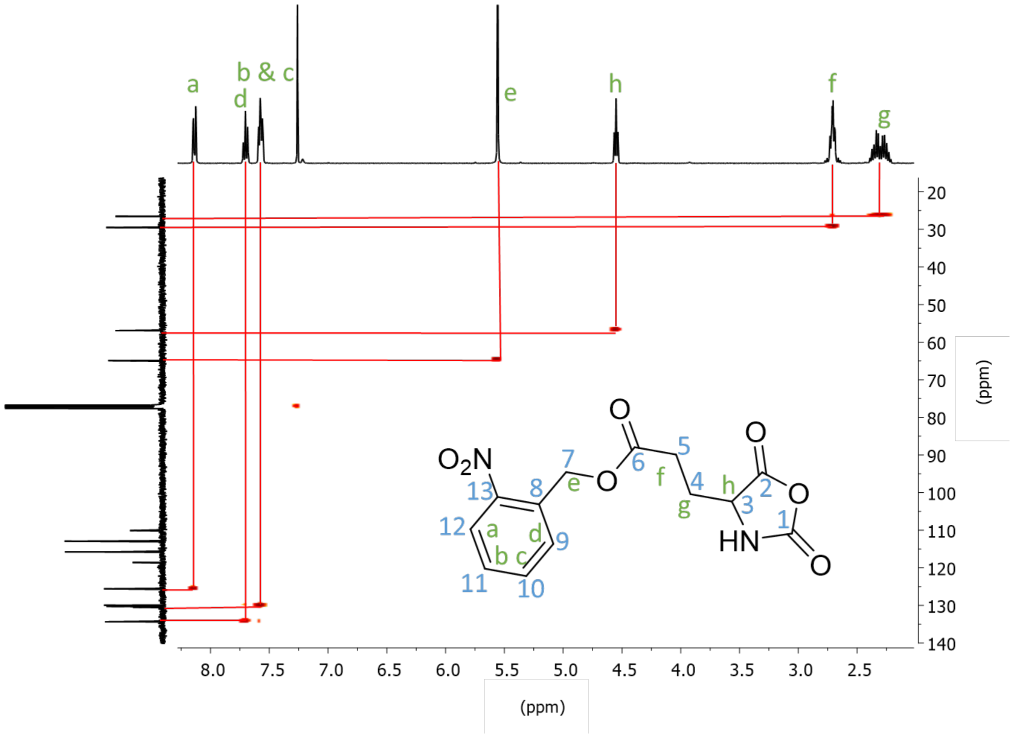

Figure S16: ^{1}H-^{13}C HSQC NMR spectra ($CDCl_3$/d-TFA (1:1)) of oNB-Glutamate NCA including assignments.

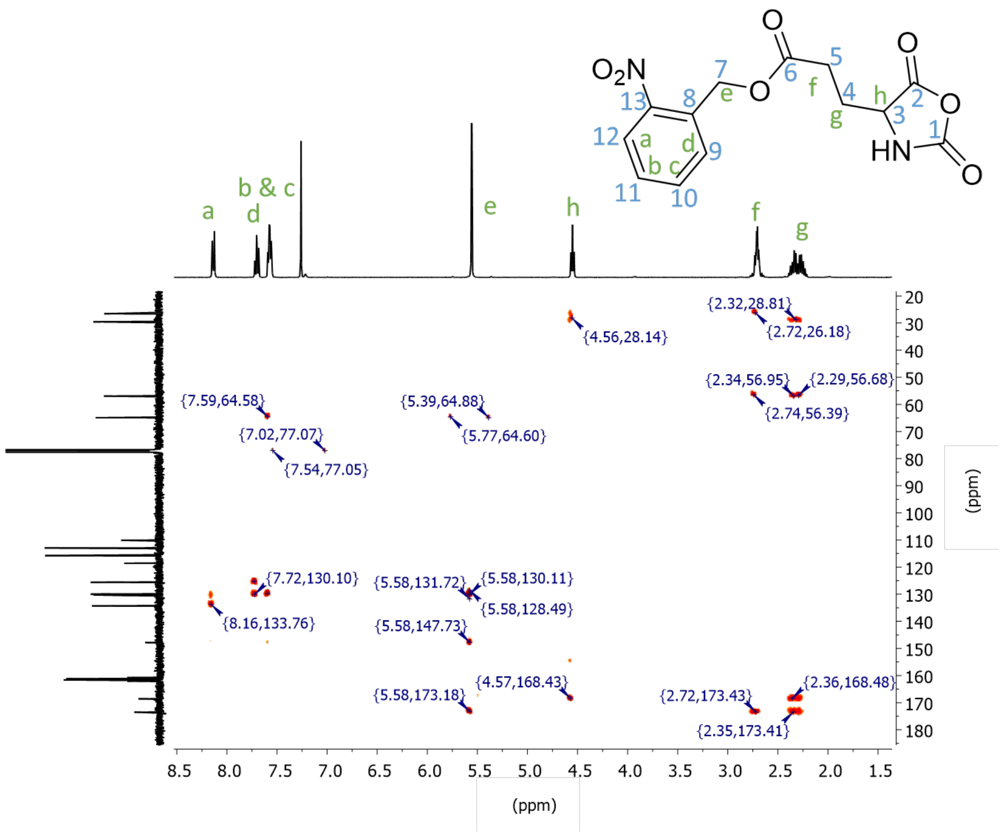

Figure S17: ^{1}H-^{13}C HMBC NMR spectra ($CDCl_3$/d-TFA (1:1)) of oNB-Glutamate NCA including assignments.

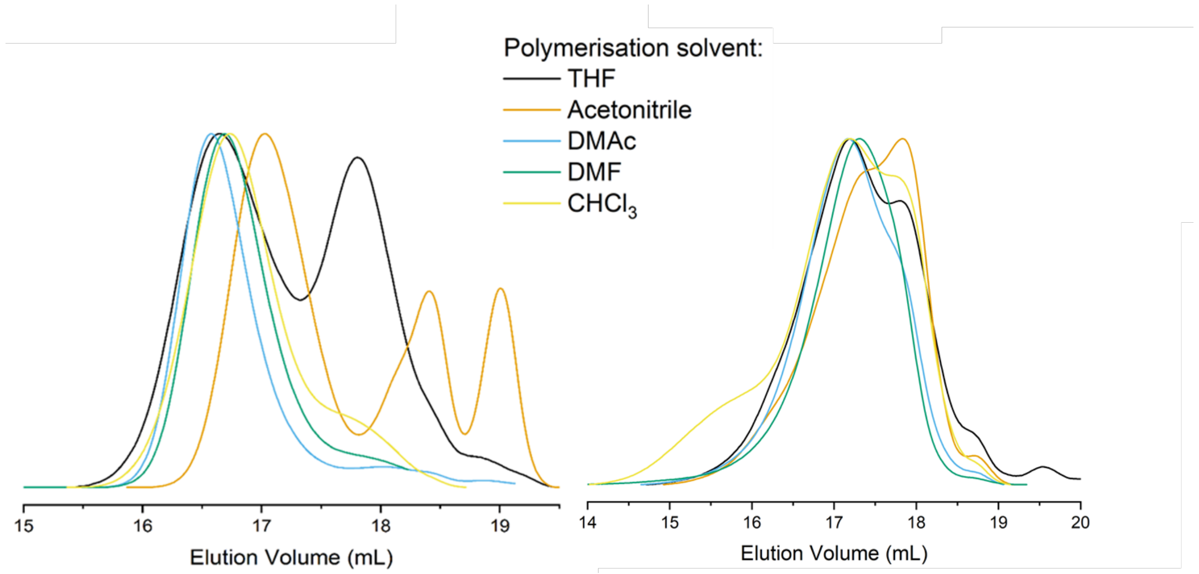

Figure S18: HFiP SEC traces of P(oNB-Glu) (left) and P(oNB-Cys) (right) of polypeptides at a targeted DP of 25 varying by the solvent in which the polymerizations occurred in (initiator butylamine).

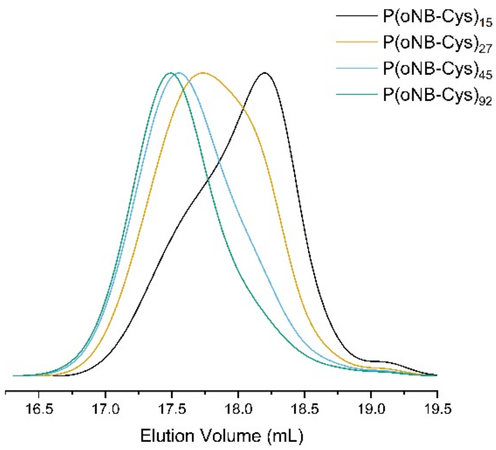

Figure S19: Size Exclusion Chromatography (SEC) traces of p(oNB-Cys) homopolypeptides with different targeted degree of polymerization (DP) using HFiP as the eluent.

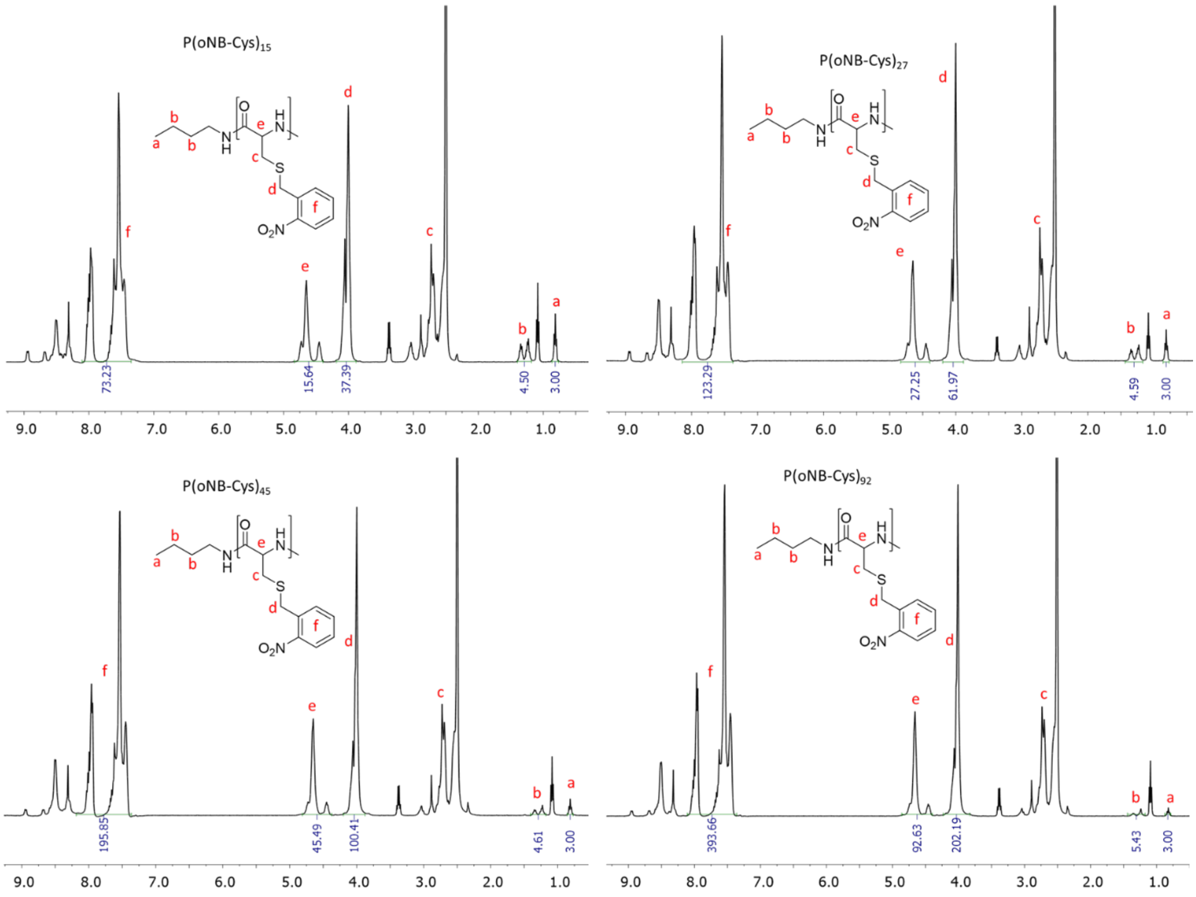

Figure S20: ^{1}H NMR of the homopolypeptides of P(oNB-Cys) which was used to calculate DP based on end group analysis of the terminal CH_3 of butylamine labelled a.

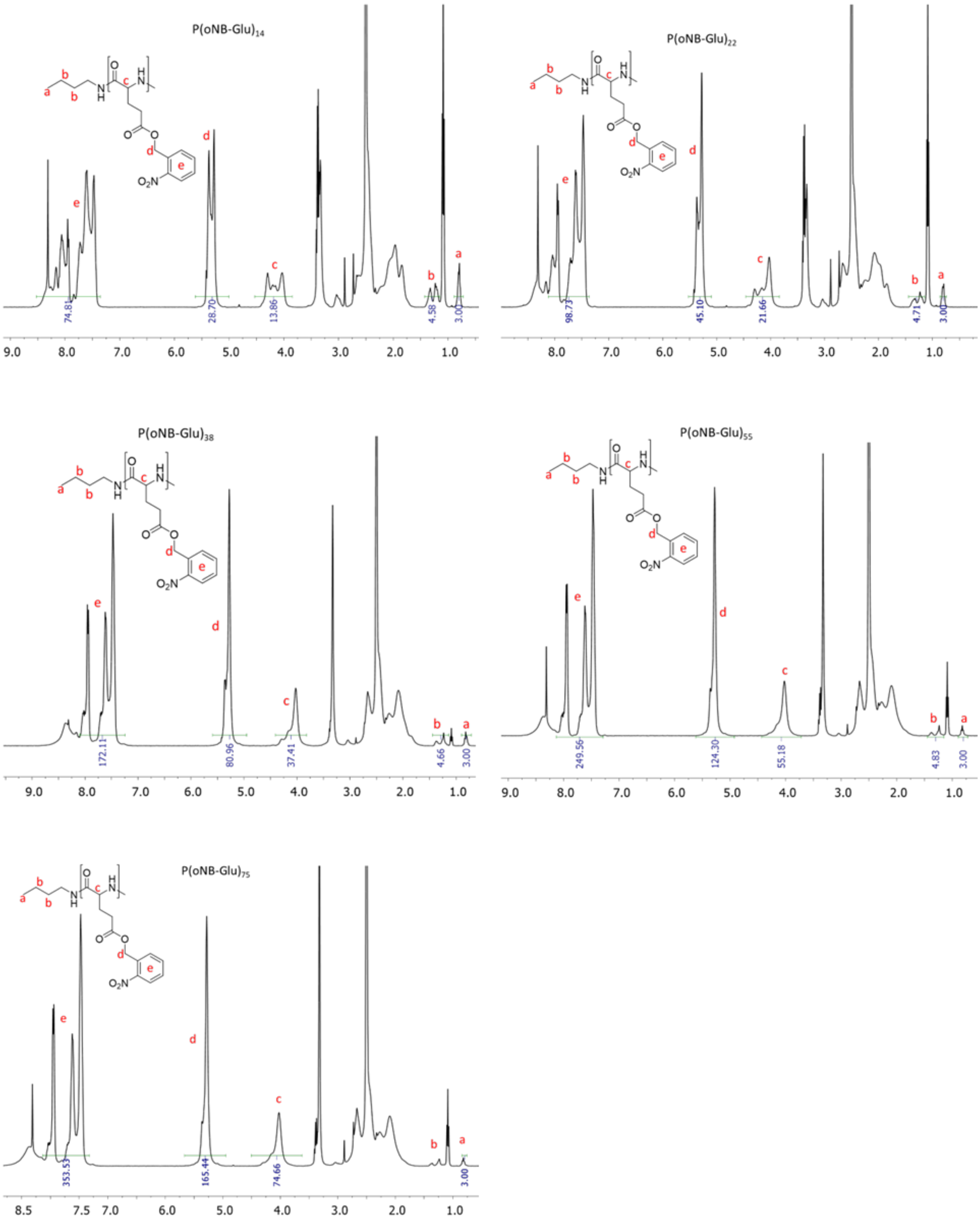

Figure S21: ^{1}H NMR of the homopolypeptides of P(oNB-Glu) which was used to calculate DP based on end group analysis of the terminal CH_3 of butylamine labelled a.

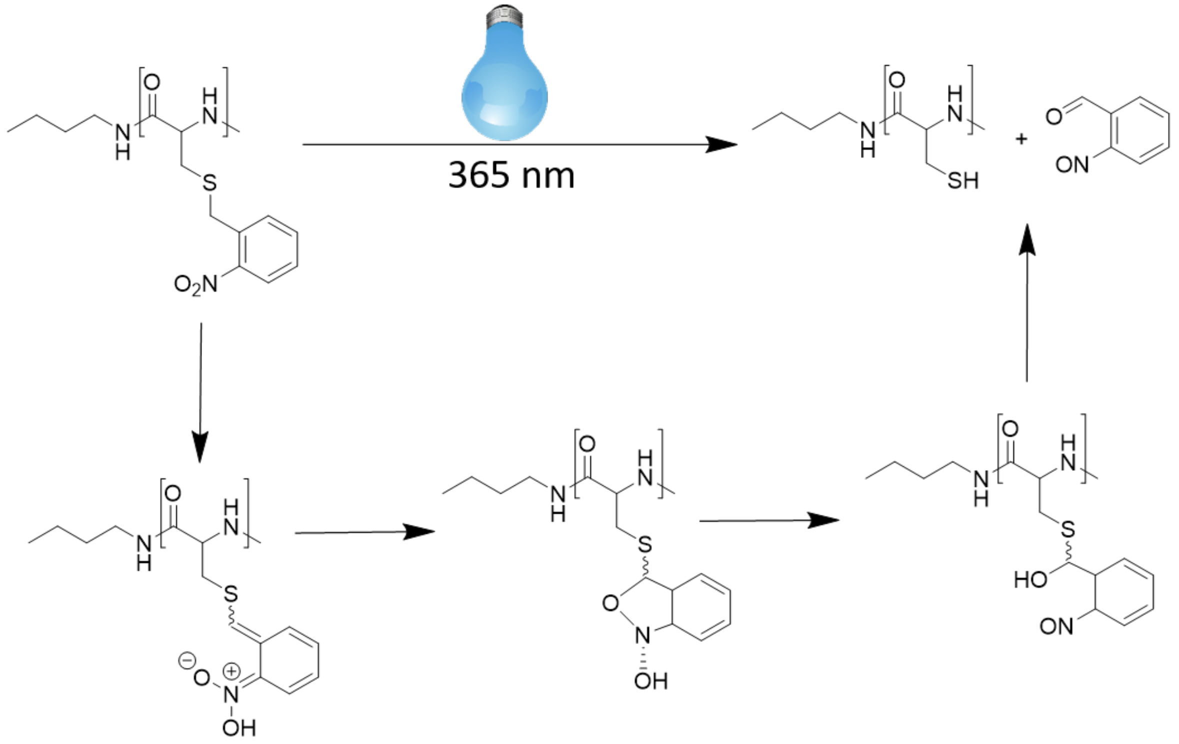

Scheme S22: Mechanism of deprotection of P(oNB-Cys) under 365 nm light revealing P(Cys) and *o*-nitrosbenzaldehyde as the by-product.[1]

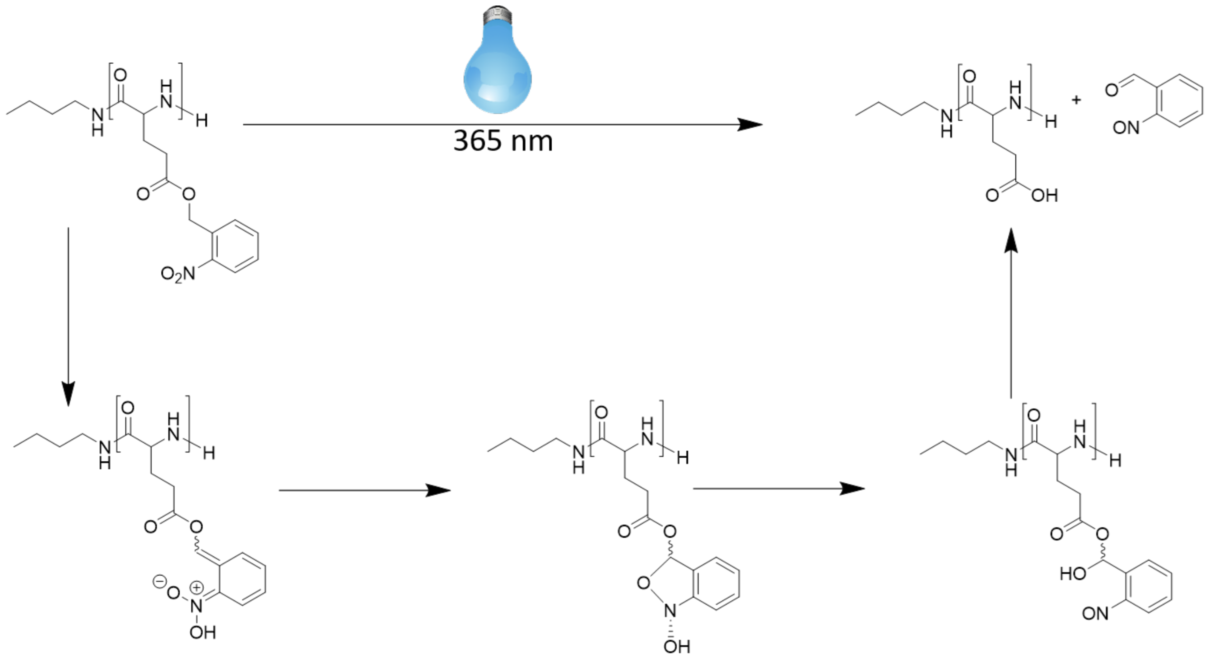

Scheme S23: Mechanism of deprotection of P(oNB-Glu) under 365nm light revealing P(Glu) and *o*-nitrosbenzaldehyde as the by-product.

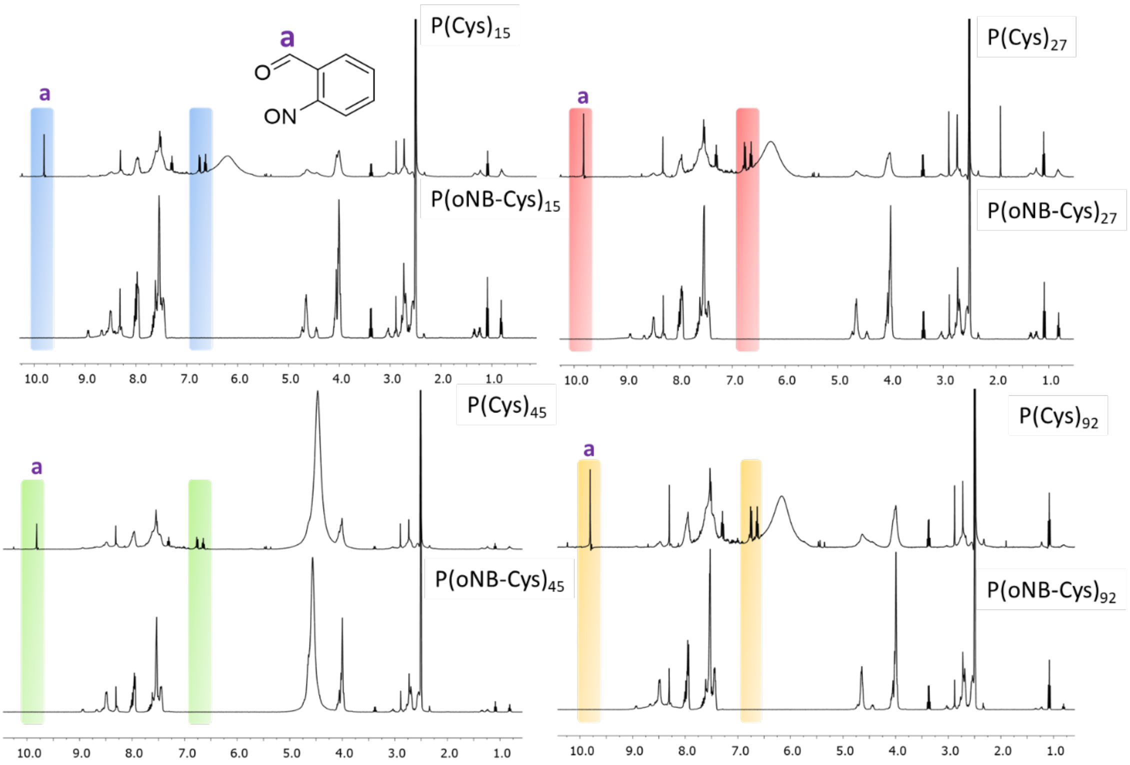

Figure S24: ^{1}H NMR spectra of P(Cys) series protected (bottom) compared to after UV-exposed (top) (d_6-DMSO) displaying the appearance of nitrosobenzaldehyde at 9.8 ppm (a).

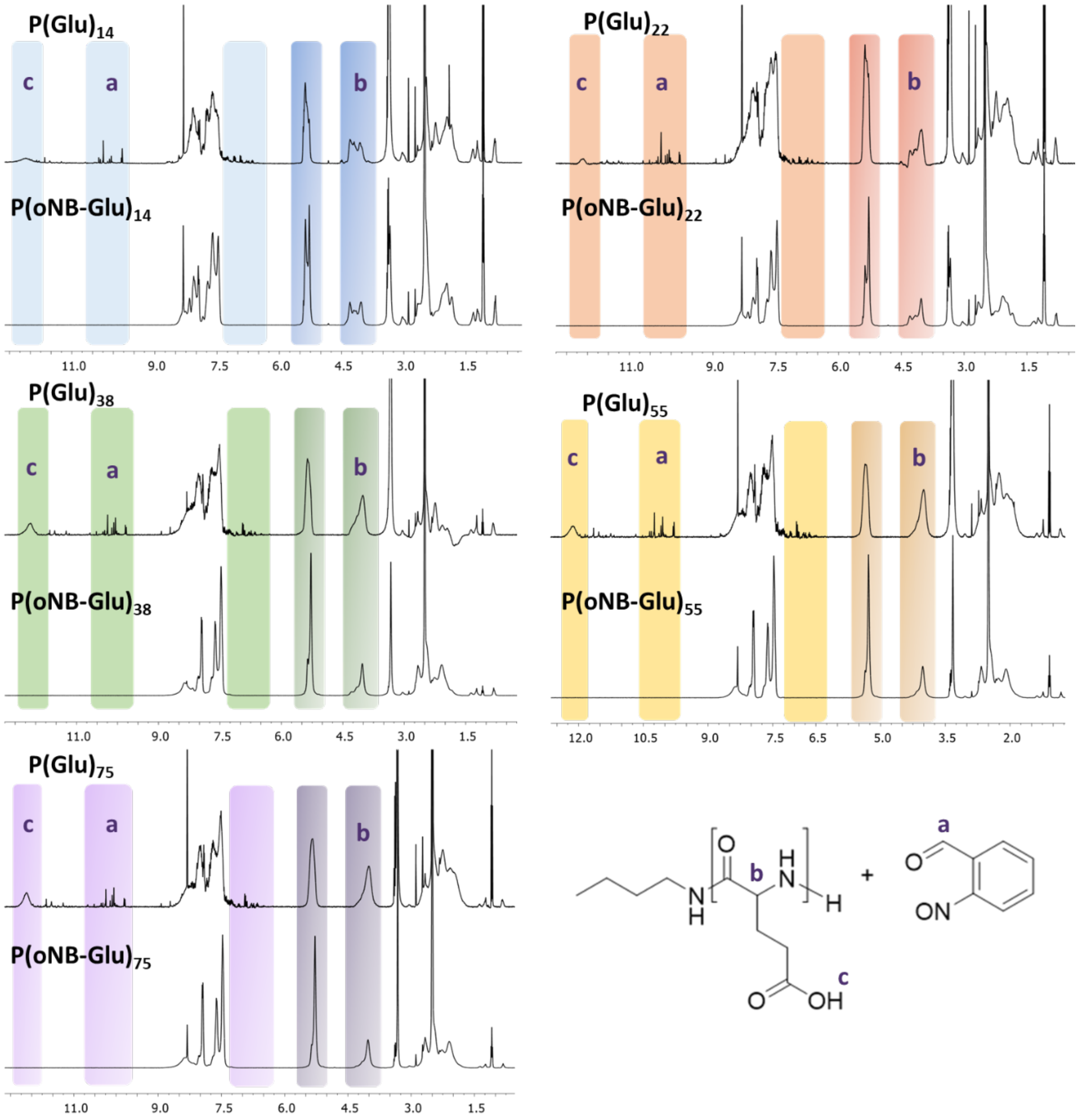

Figure S25: ^{1}H NMR spectra of P(Glu) series protected (bottom) compared to after UV-exposed (top) (d_6-DMSO) displaying the appearance of nitrosobenzaldehyde at 9.8 ppm (a) and backbone (b) and carboxylic acid (c) of P(Glu).

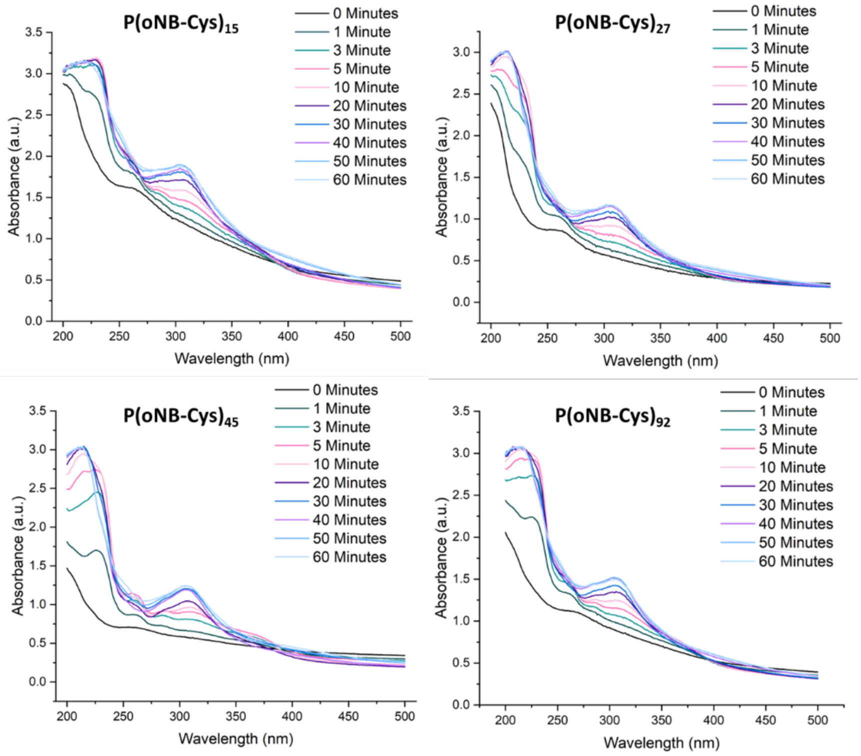

Figure S26: UV-Vis traces for P(oNB-Cys) homopolypeptides series at consecutive time points over the 60 minutes cumulative curing under 365nm light.

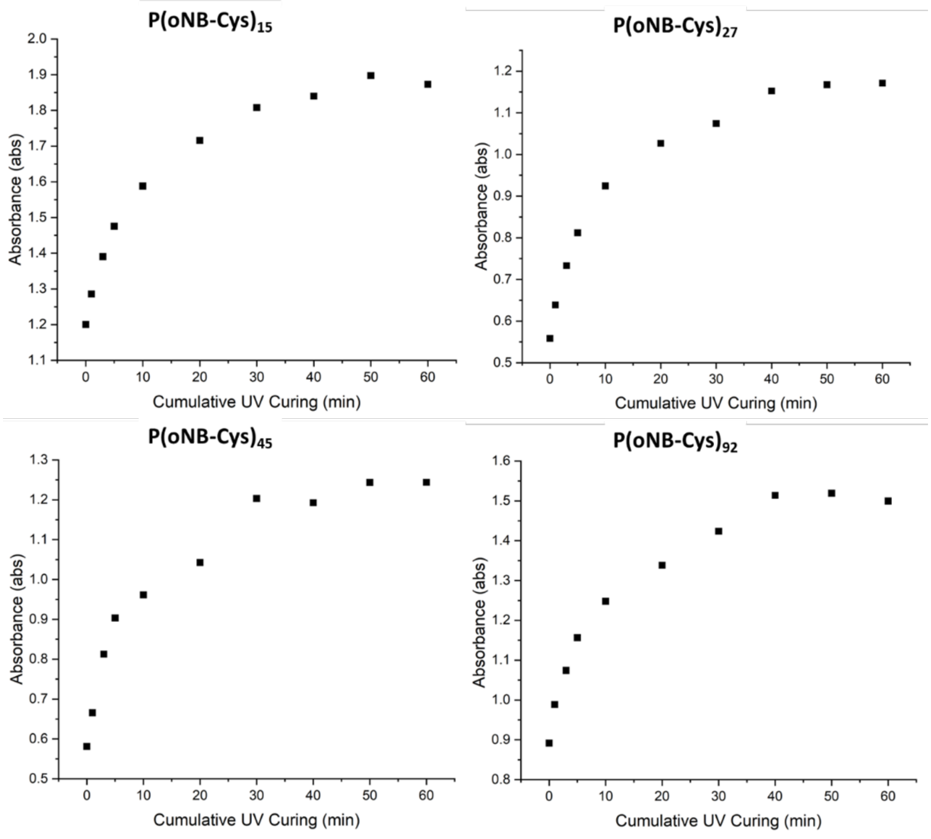

Figure S27: Absorbance at 305nm for P(oNB-Cys) homopolypeptide series at consecutive time points over the 60 minutes cumulative curing under 365nm light.

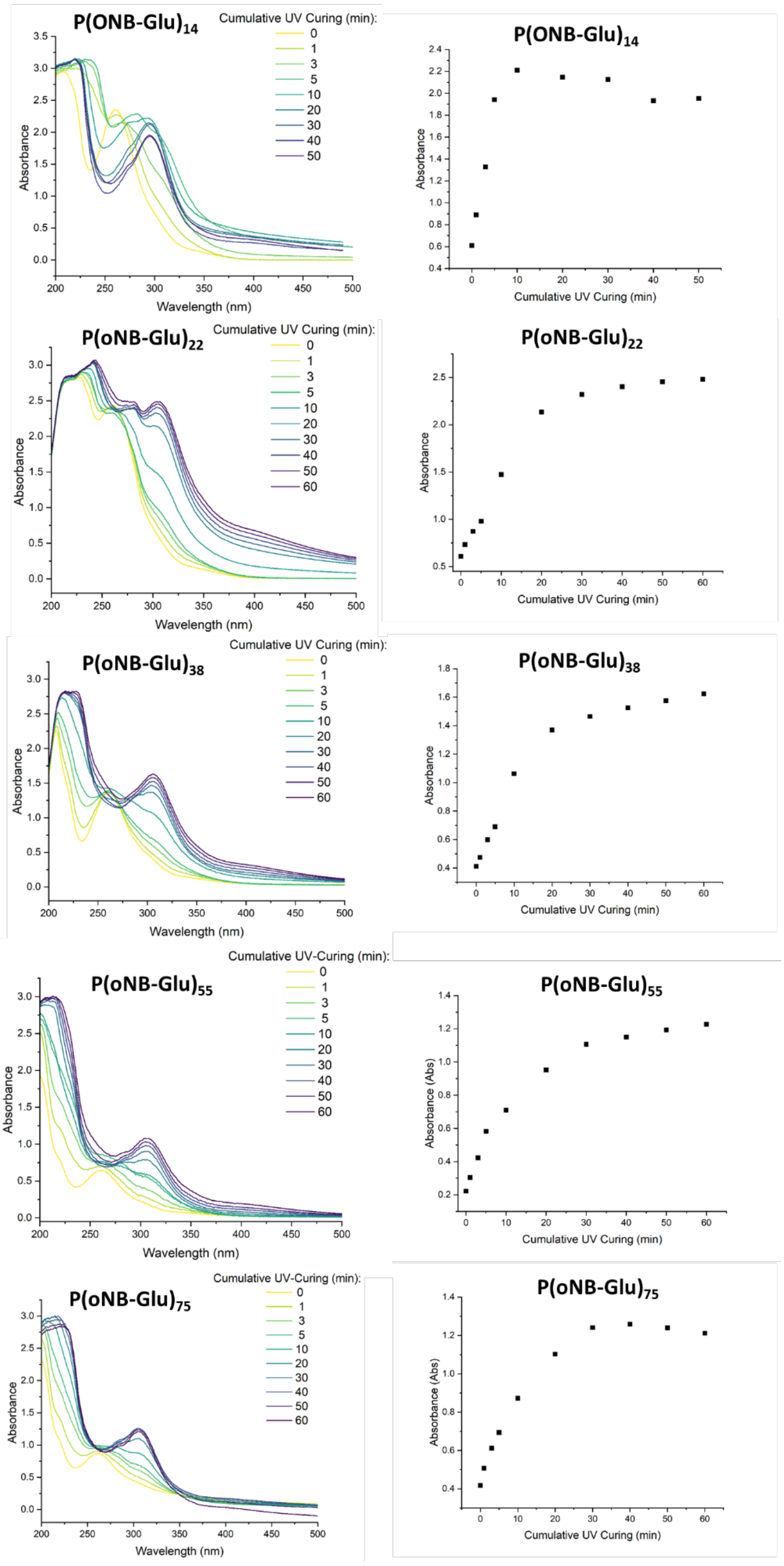

Figure S28: UV-Visible spectra of P(oNB-Glu) homopolypeptide series at consecutive time points over the 60 minutes cumulative curing under 365nm light (left) and the absorbance at 305nm against cumulative curing time (right).

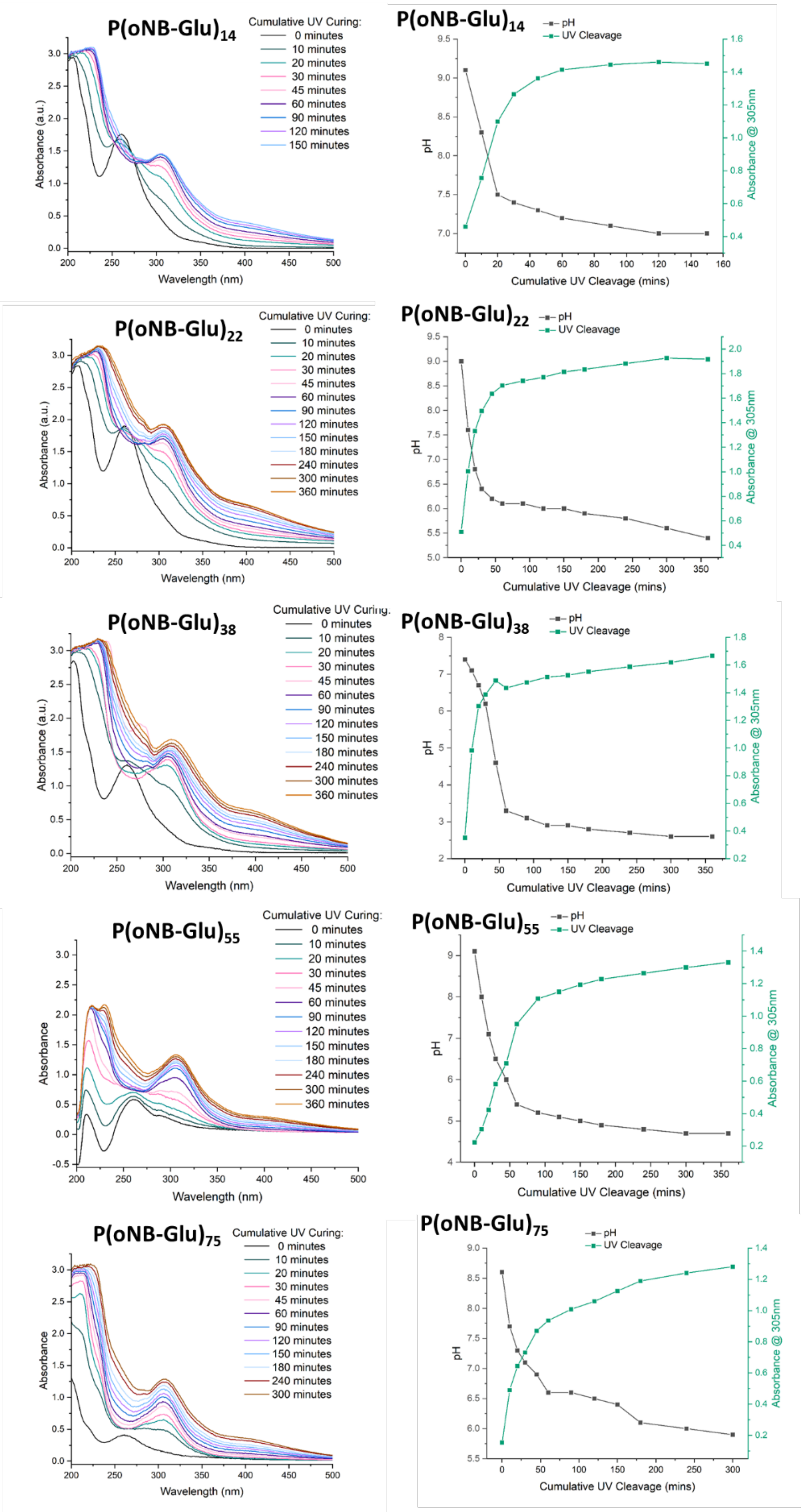

Figure S29: UV-Visible spectra of P(oNB-Glu) homopolypeptide series at consecutive time points over the 300 minutes cumulative curing under 365nm light (left) and the absorbance at 305nm and change in pH against cumulative curing time (right).

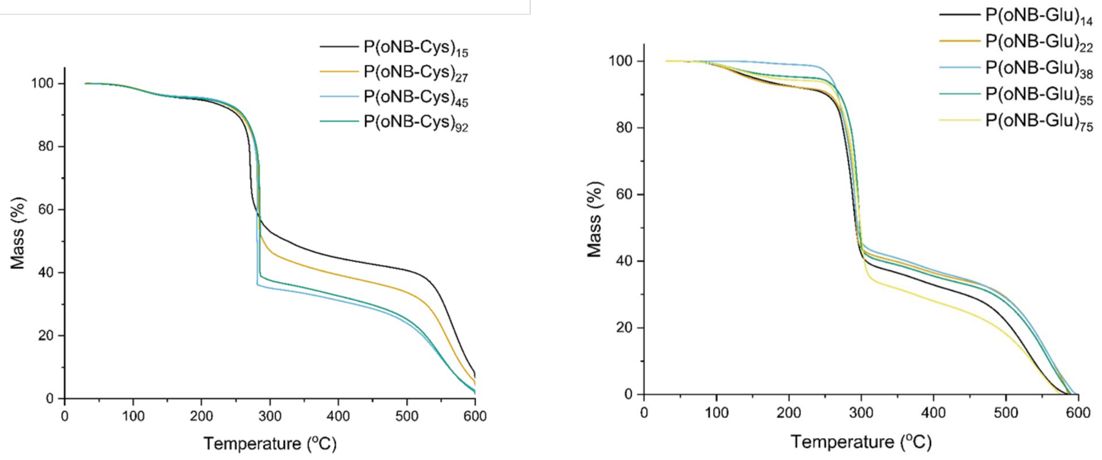

Figure S30: Thermogravimetric analysis (TGA) traces of the different homopolypeptides (P(oNB-Cys)) (left) and P(oNB-Glu)) (right).

NCA was added to give P(Z-L-Lys)-b-P(L-Phe) (3). Acidic deprotection, TFA and 33% HBr in acetic acid (4) and subsequent glycosylation with lactobionic acid (5).

Table S2: Z-Average diameters as reported by DLS for each time point.

**Monomer(s)**		**% by mass**		**24hrs [a]**		**Dialysis [b]**		**UV exposed [c]**		**Dialysis post UV exposure[d]**	
Glu Variant	Cys Variant	% Glu	% Cys	Size [nm]	PDI	Size [nm]	PDI	Size [nm]	PDI	Size [nm]	PDI
Bn-Glu	-	100	-	102	0.19	109	0.19	-	-	-	-
Bn-Glu	oNB-Cys	75	25	117	0.19	129	0.19	129	0.19	126	0.18
Bn-Glu	oNB-Cys	50	50	130	0.14	137	0.13	139	0.14	137	0.14
Bn-Glu	oNB-Cys	25	75	136	0.11	142	0.10	137	0.11	142	0.11
-	oNB-Cys		100	148	0.09	157	0.10	154	0.12	157	0.10
oNB-Glu	oNB-Cys	25	75	136	0.11	142	0.10	141	0.10	141	0.11
oNB-Glu	oNB-Cys	50	50	126	0.13	128	0.11	130	0.12	129	0.12
oNB-Glu	oNB-Cys	75	25	120	0.12	124	0.11	123	0.11	123	0.12
oNB-Glu	-	100	-	120	0.16	125	0.16	121	0.17	115	0.15
oNB-Glu	Bn-Cys	75	25	130	0.09	135	0.10	124	0.11	117	0.12
oNB-Glu	Bn-Cys	50	50	137	0.14	140	0.10	134	0.12	132	0.14
oNB-Glu	Bn-Cys	25	75	160	0.22	163	0.21	160	0.21	161	0.21
-	Bn-Cys	-	100	173	0.22	172	0.22	-	-	-	-

[a] 24hrs open to air polymerisation to remove DCM, [b] 72 hrs purification by dialysis, [c] UV-exposed for ca. 360 minutes and [d] 72hrs purification by dialysis after UV-Cleavage.

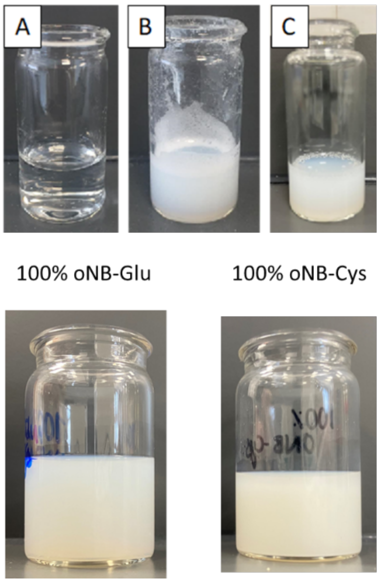

Figure S31: Images of the different stages of miniemulsion nanoparticle formation. Top A- surfactant solution (8mg mL^{-1}), B- after emulsification, C- after 24hrs open to air. Bottom nanoparticles containing photoreactive protective groups on the homopolypeptide within the core.

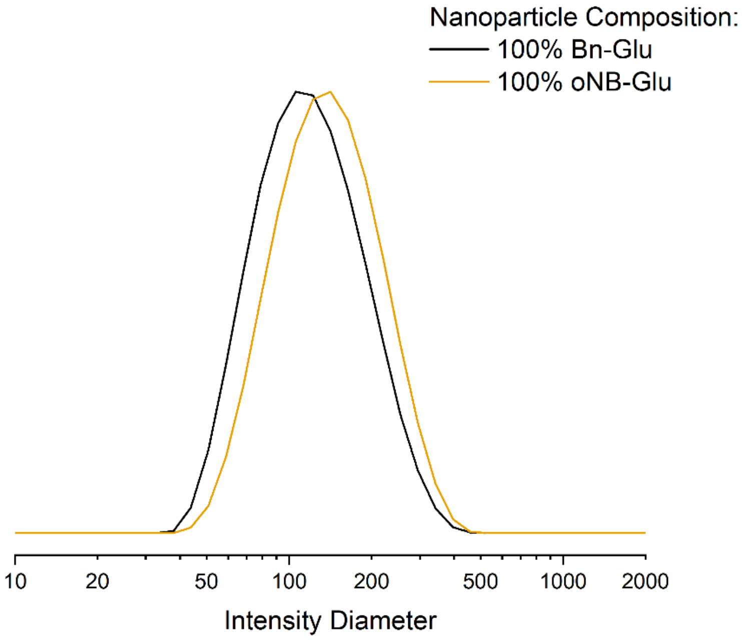

Figure S32: DLS intensity traces comparing the diameters measured after dialysis for Bn-Glu (black) and oNB-Glu (yellow).

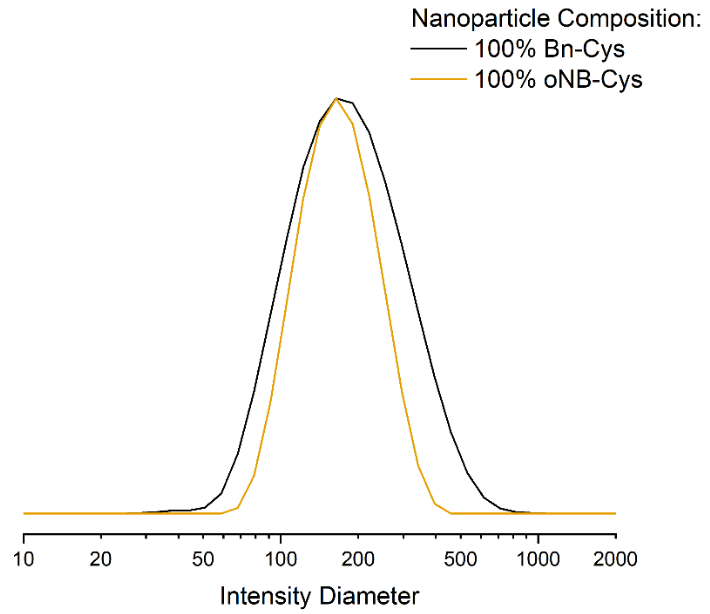

Figure S33: DLS intensity traces comparing the diameters measured after dialysis for Bn-Cys (black) and oNB-Cys (yellow).

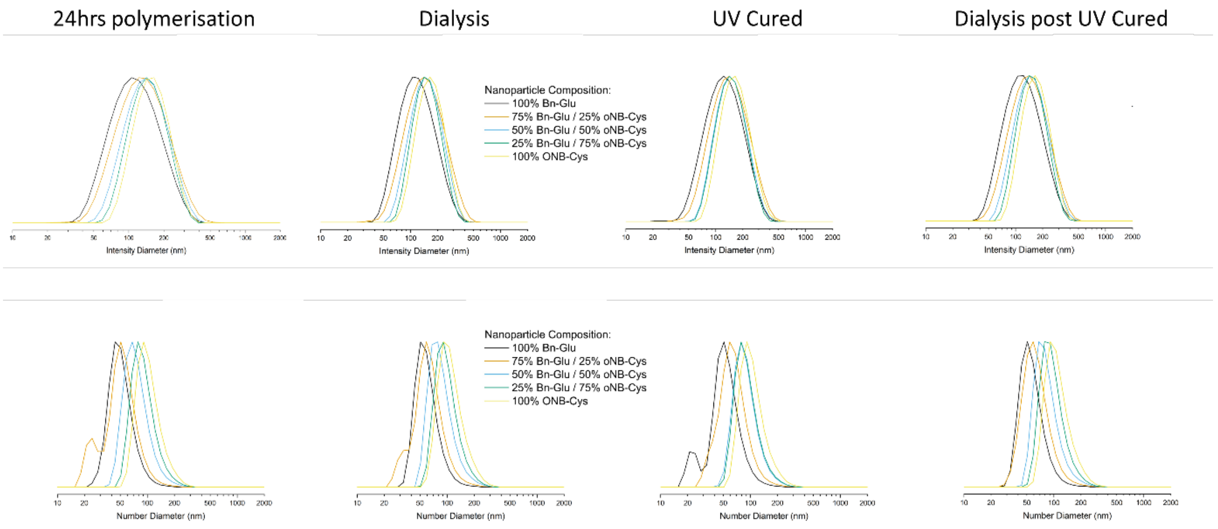

Figure S34: Intensity (top) and number (bottom) average traces for the Bn-Glu/oNB-Cys nanoparticle series synthesised across the 4 time points during synthesis and purification.

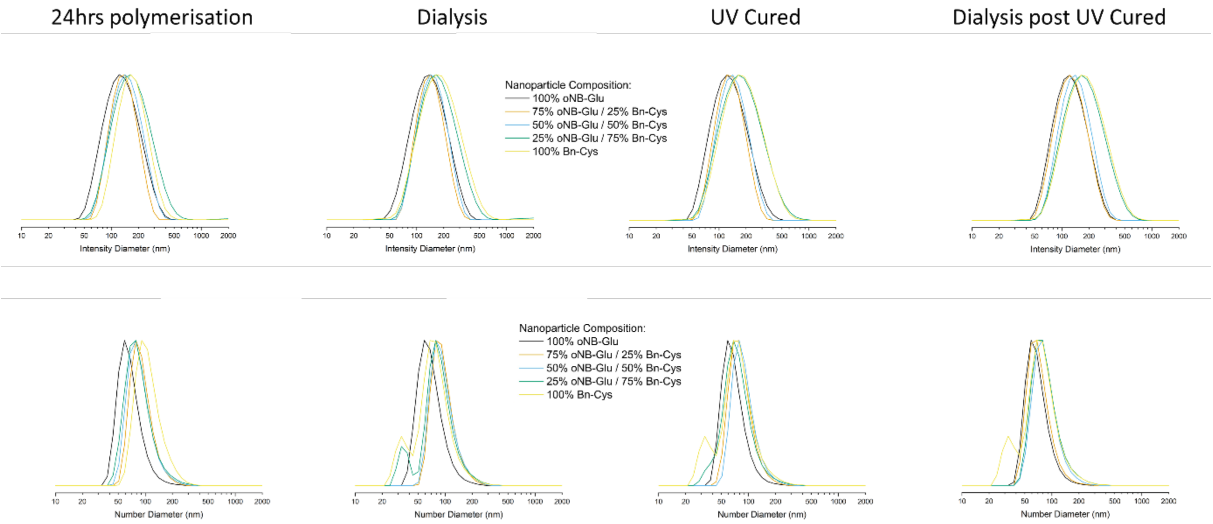

Figure S35: Intensity (top) and number (bottom) average traces for the oNB-Glu/Bn-Cys nanoparticle series synthesised across the 4 time points during synthesis and purification.

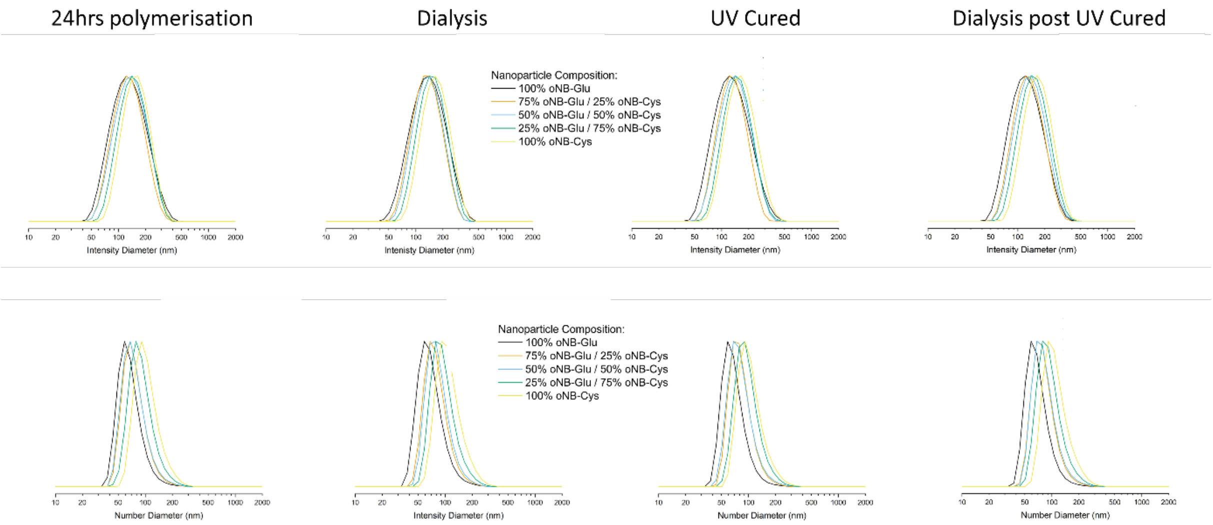

Figure S36: Intensity (top) and number (bottom) average traces for the oNB-Glu/Bn-Cys nanoparticle series synthesised across the 4 time points during synthesis and purification.

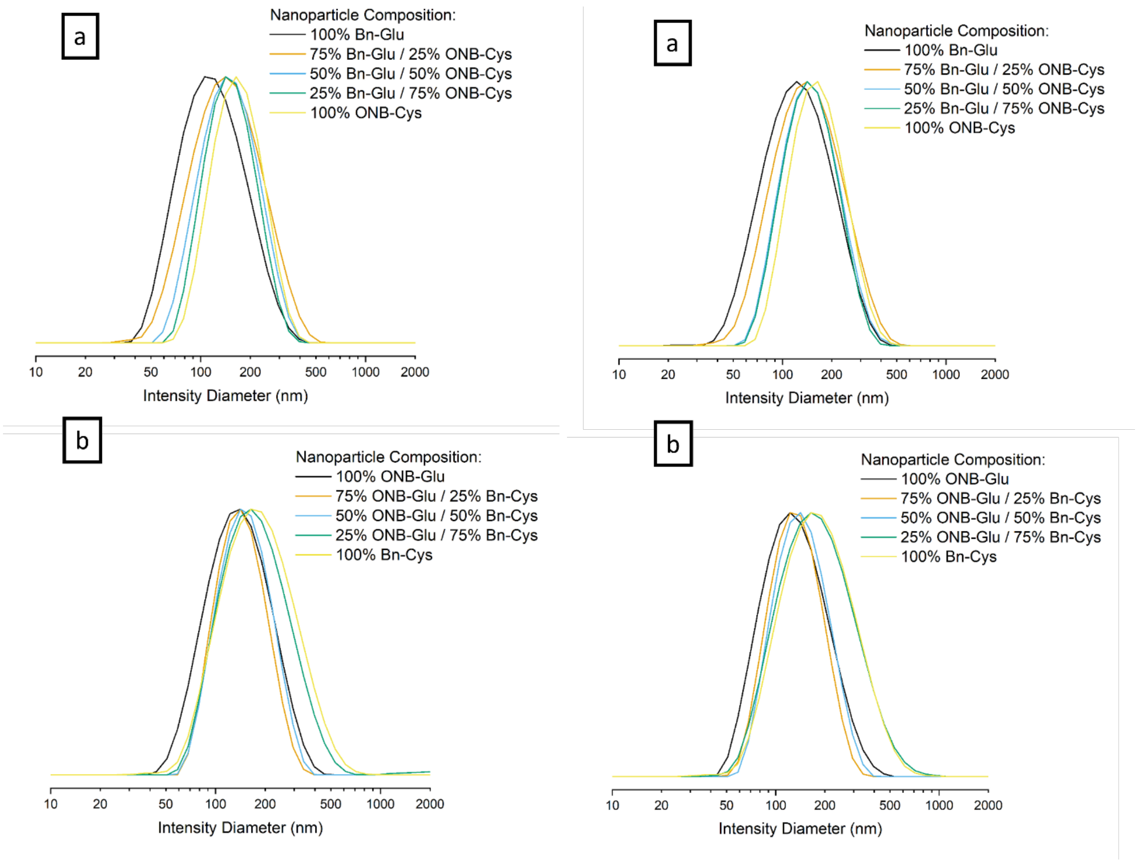

Figure S37: DLS intensity average traces after dialysis before UV-treatment for a) Bn-Glu/oNB-Cys and b) oNB-Glu/Bn-Cys nanoparticle series. DLS intensity average traces after dialysis post UV-treatment a) Bn-Glu/oNB-Cys and b) oNB-Glu/Bn-Cys nanoparticle series.

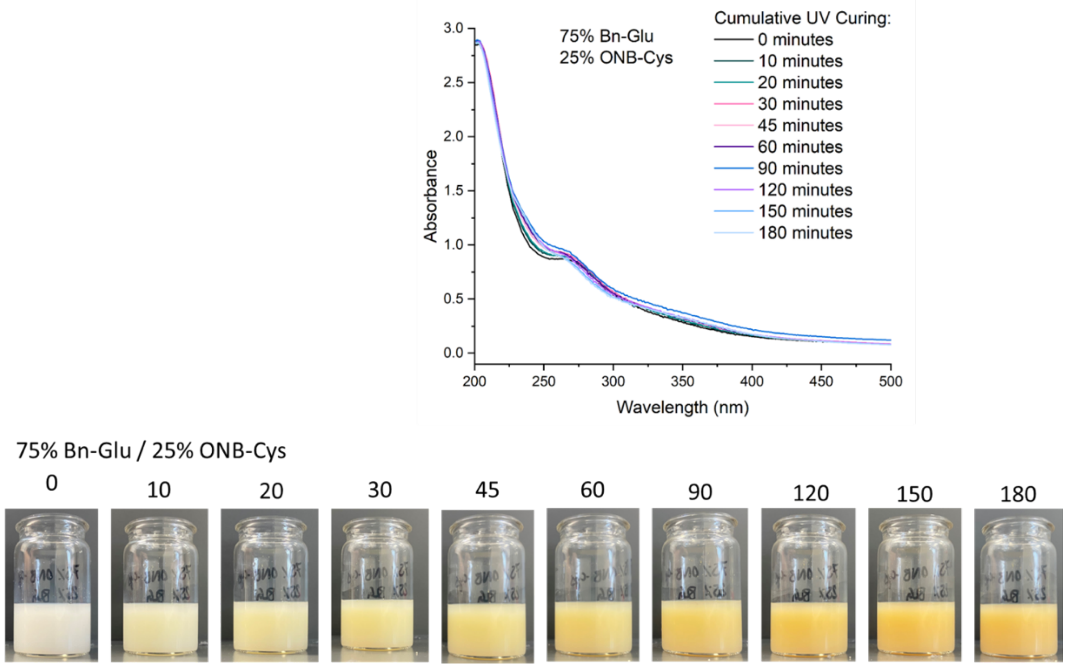

Figure S38: UV-Vis spectra in H_2O of the cleavage under 365nm light of emulsion nanoparticles with initial monomer composition of 75% Bn-Glu and 25% oNB-Cys (top) and the images of the nanoparticle bulk solution at each time point (bottom).

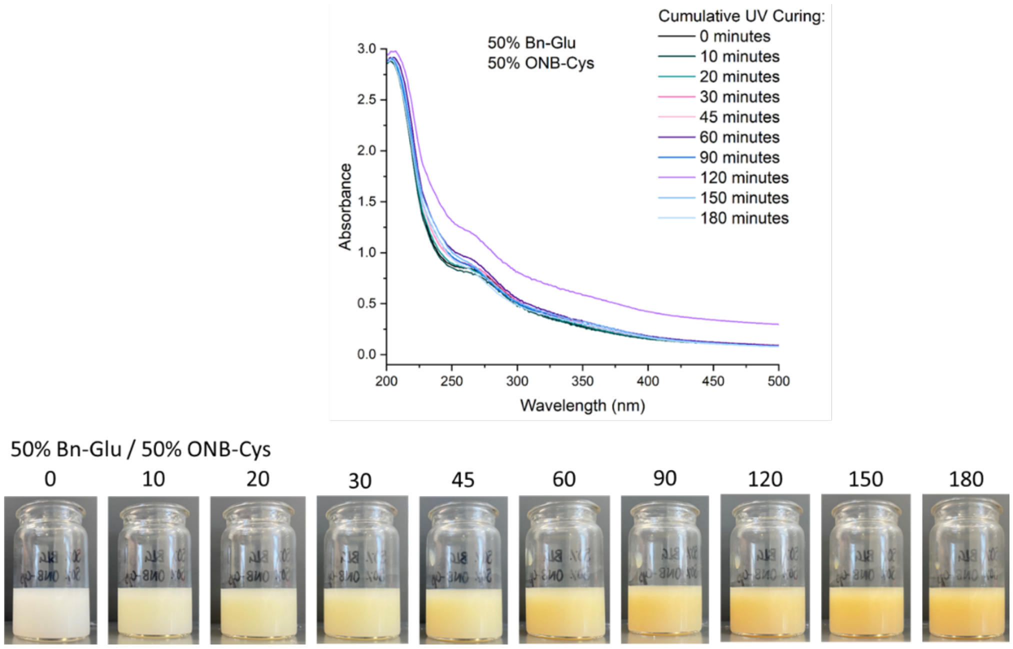

Figure S39: UV-Vis spectra in H_2O of the cleavage under 365nm light of emulsion nanoparticles with initial monomer composition of 50% Bn-Glu and 50% oNB-Cys (top) and the images of the nanoparticle bulk solution at each time point (bottom).

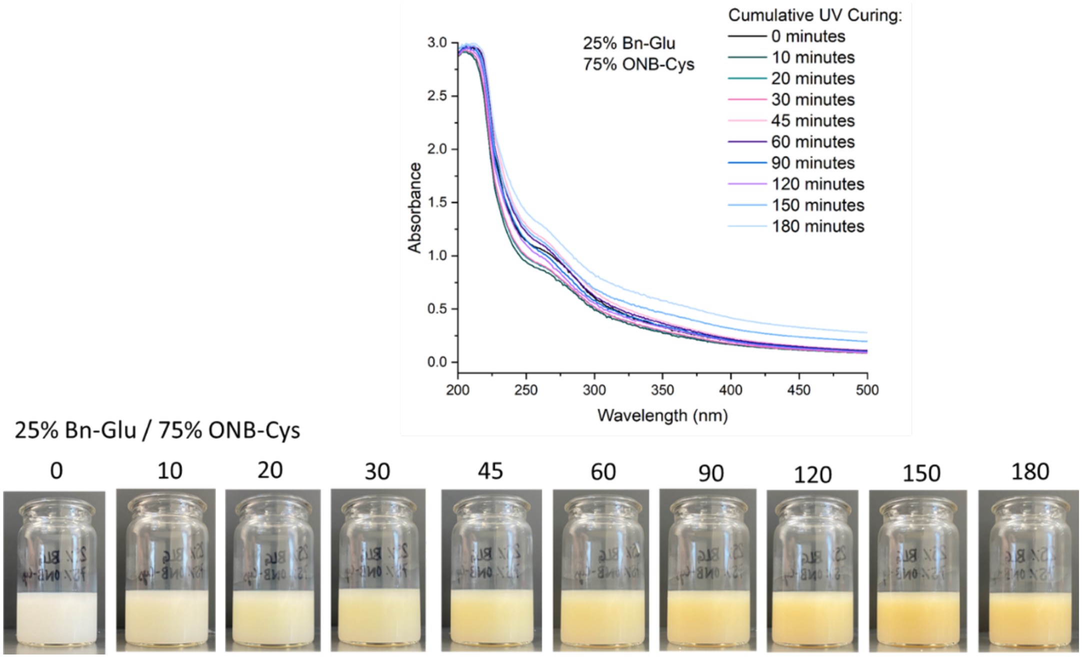

Figure S40: UV-Vis spectra in H_2O of the cleavage under 365nm light of emulsion nanoparticles with initial monomer composition of 25% Bn-Glu and 75% oNB-Cys (top) and the images of the nanoparticle bulk solution at each time point (bottom).

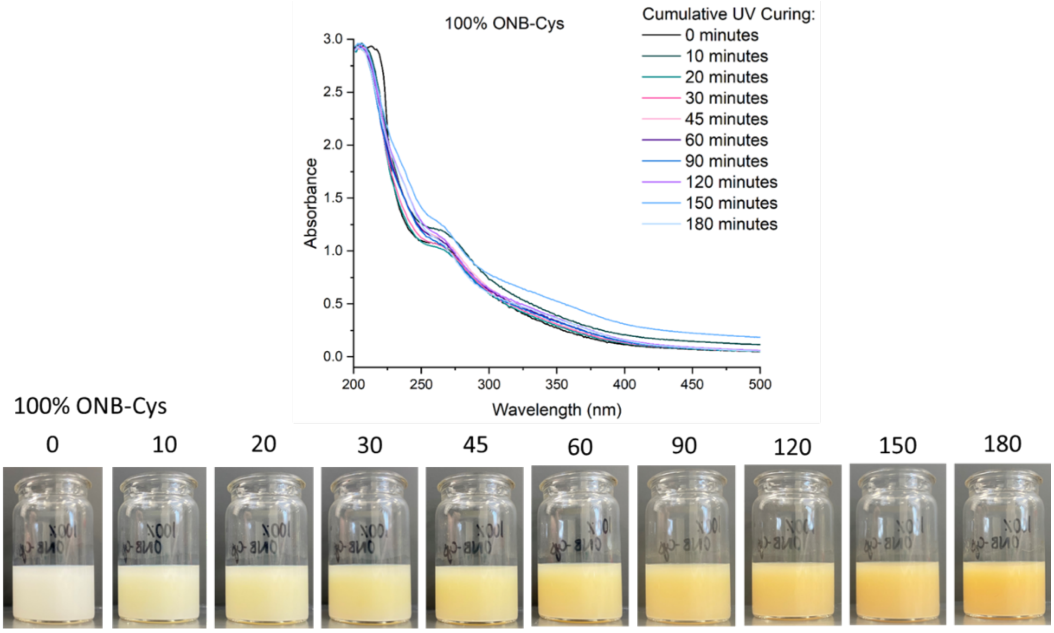

Figure S41: UV-Vis spectra in H_2O of the cleavage under 365nm light of emulsion nanoparticles with initial monomer composition of 100% oNB-Cys (top) and the images of the nanoparticle bulk solution at each time point (bottom).

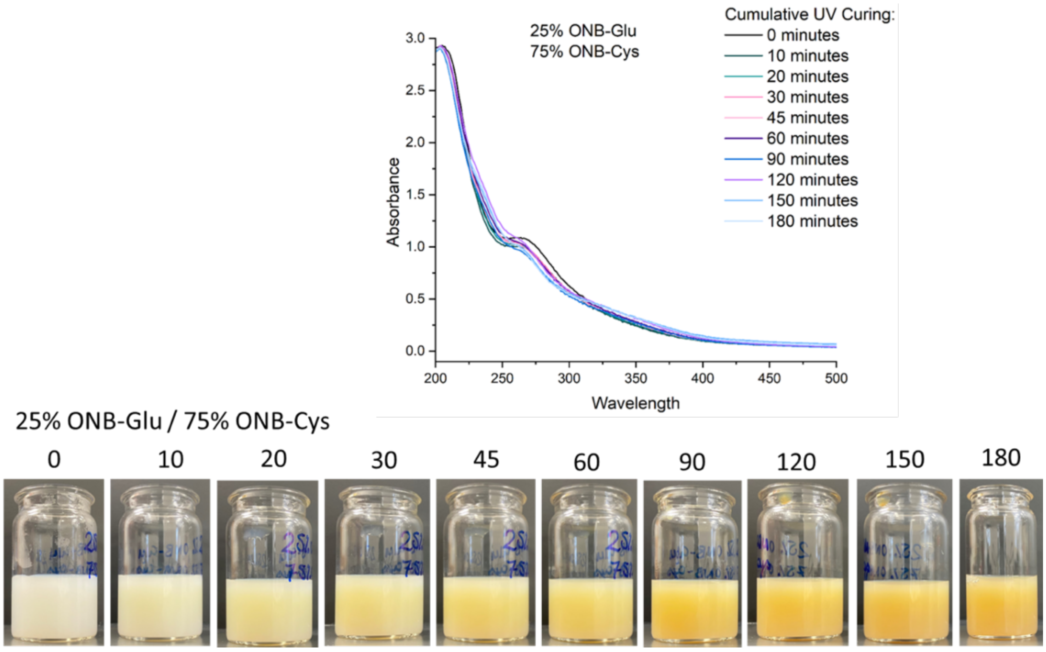

Figure S42: UV-Vis spectra in H_2O of the cleavage under 365nm light of emulsion nanoparticles with initial monomer composition of 25% oNB-Glu and 75% oNB-Cys (top) and the images of the nanoparticle bulk solution at each time point (bottom).

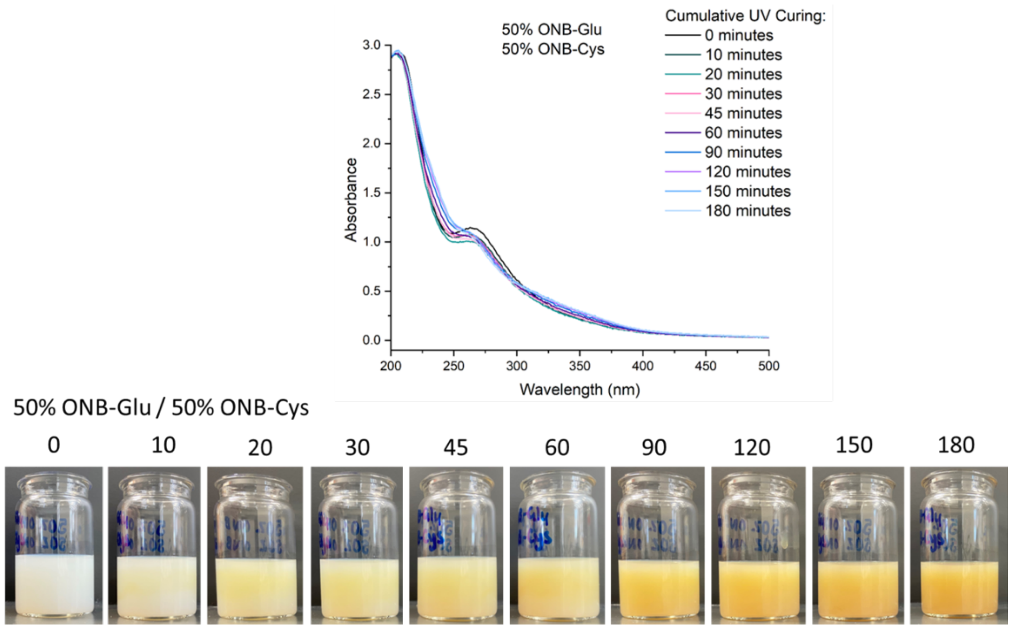

Figure S43: UV-Vis spectra in H_2O of the cleavage under 365nm light of emulsion nanoparticles with initial monomer composition of 50% oNB-Glu and 50% oNB-Cys (top) and the images of the nanoparticle bulk solution at each time point (bottom).

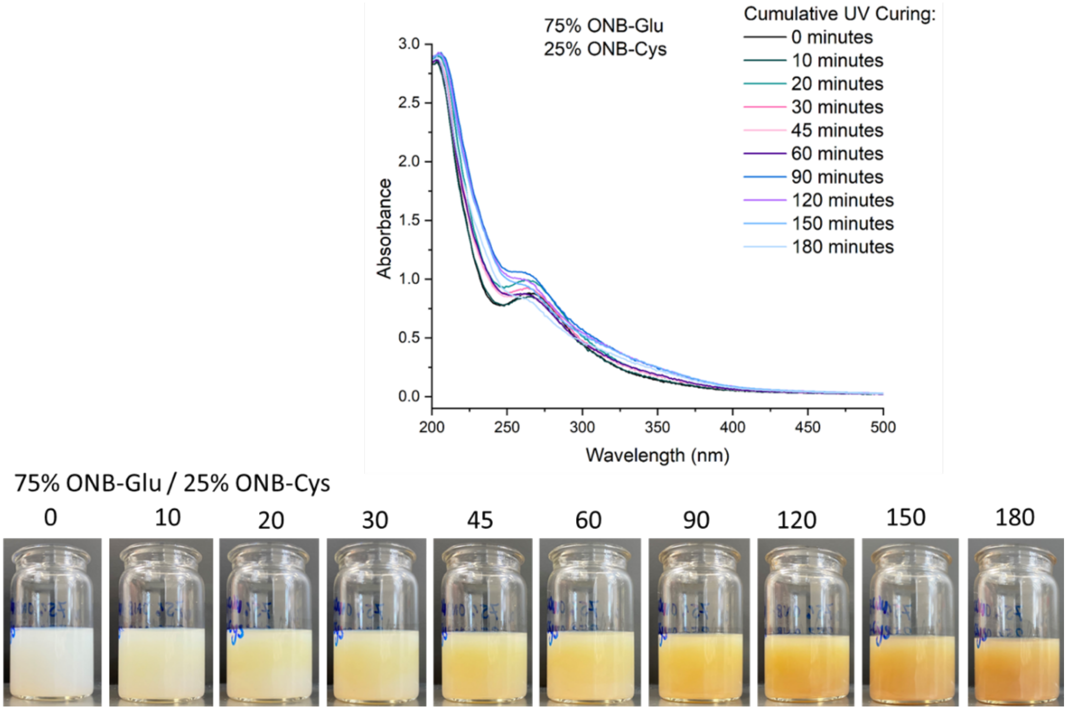

Figure S44: UV-Vis spectra in H_2O of the cleavage under 365nm light of emulsion nanoparticles with initial monomer composition of 75% oNB-Glu and 25% oNB-Cys (top) and the images of the nanoparticle bulk solution at each time point (bottom).

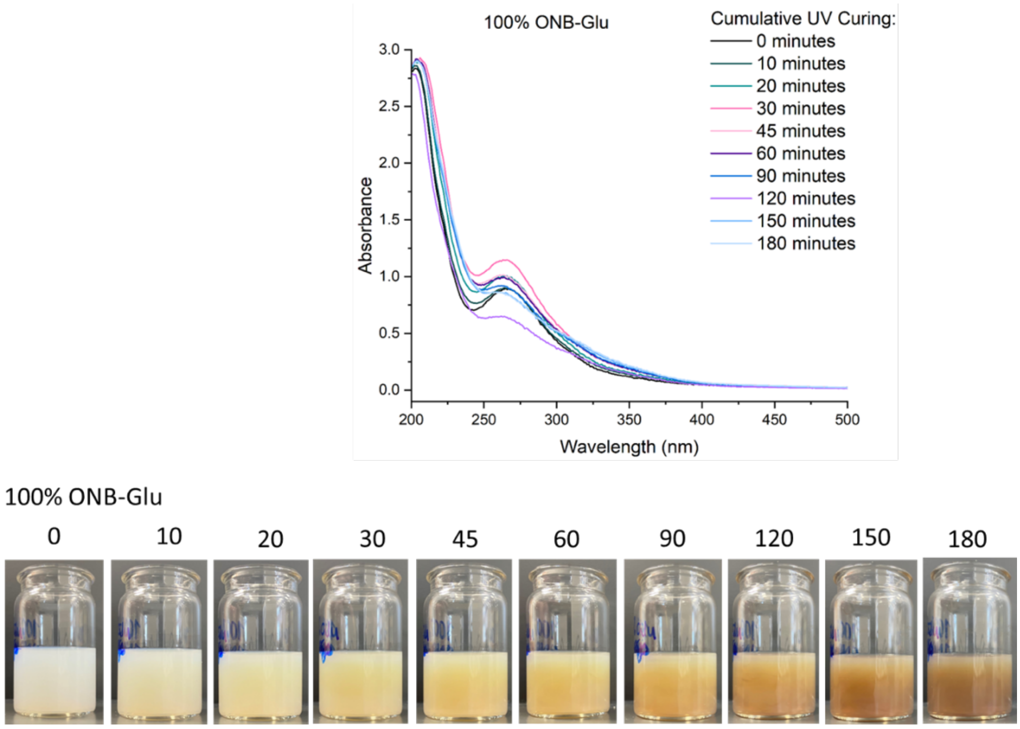

Figure S45: UV-Vis spectra in H_2O of the cleavage under 365nm light of emulsion nanoparticles with initial monomer composition of 100% oNB-Glu (top) and the images of the nanoparticle bulk solution at each time point (bottom).

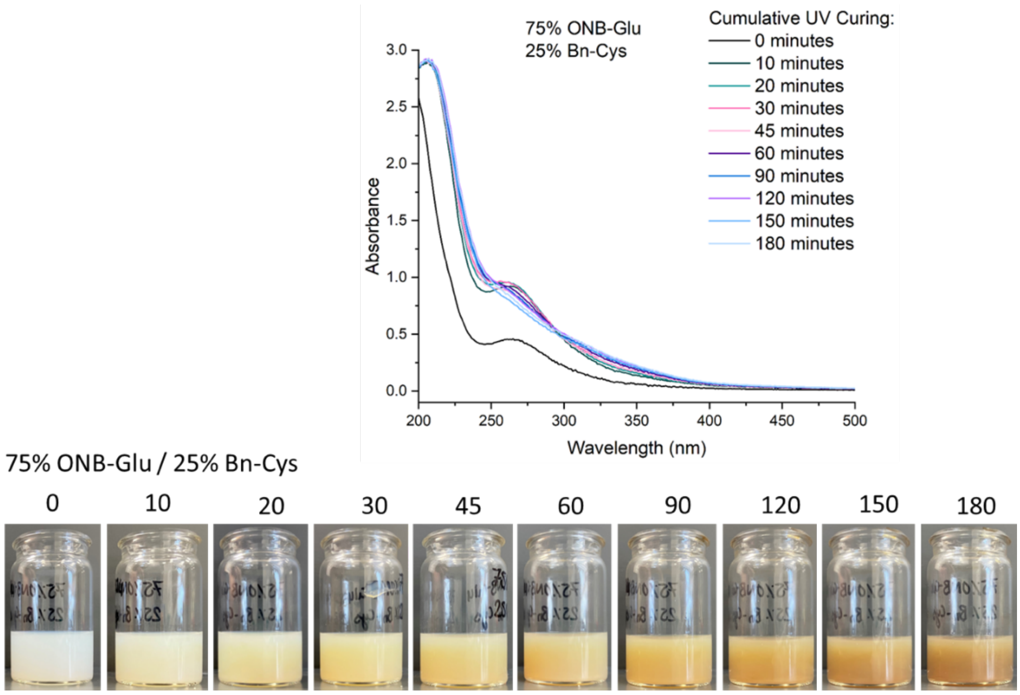

Figure S46: UV-Vis spectra in H_2O of the cleavage under 365nm light of emulsion nanoparticles with initial monomer composition of 75% oNB-Glu and 25% Bn-Cys (top) and the images of the nanoparticle bulk solution at each time point (bottom).

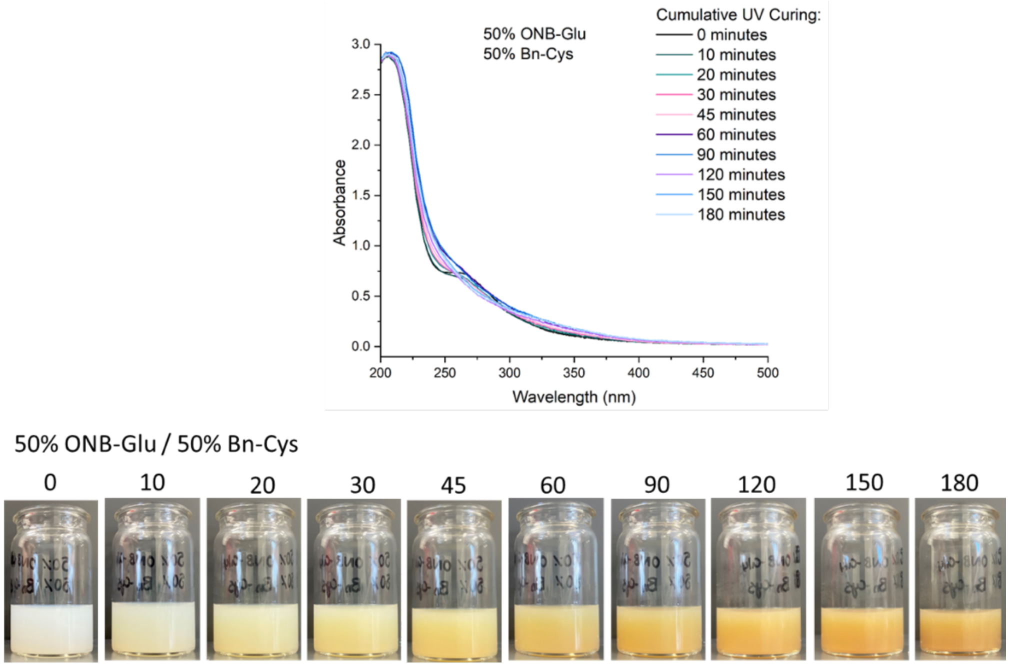

Figure S47: UV-Vis spectra in H_2O of the cleavage under 365nm light of emulsion nanoparticles with initial monomer composition of 50% oNB-Glu and 50% Bn-Cys (top) and the images of the nanoparticle bulk solution at each time point (bottom).

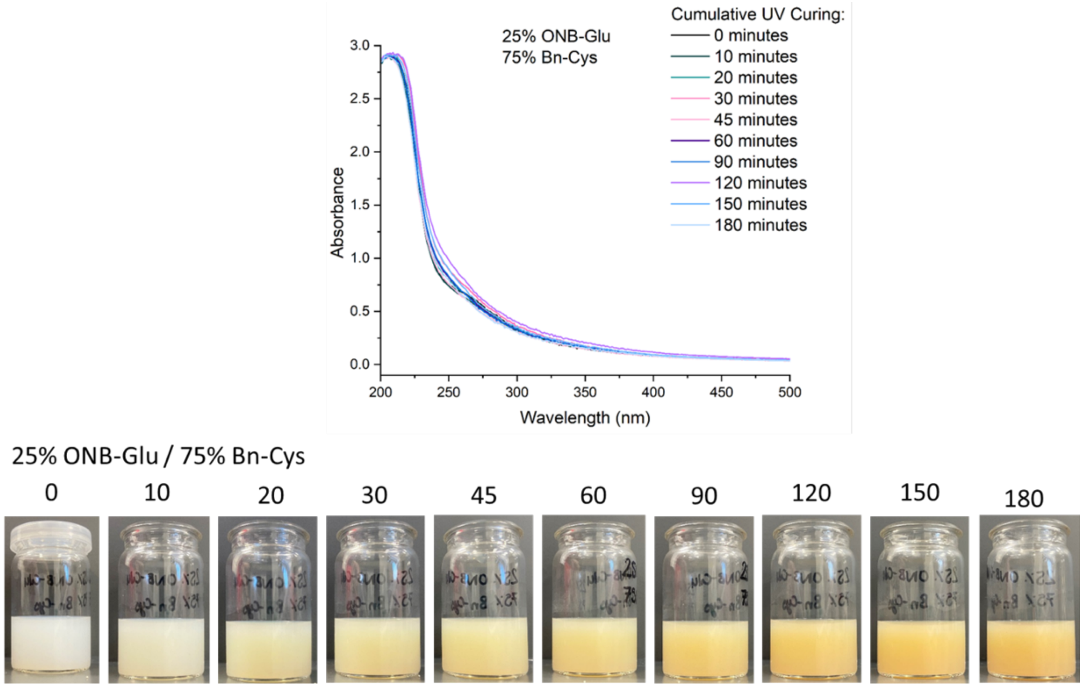

Figure S48: UV-Vis spectra in H_2O of the cleavage under 365nm light of emulsion nanoparticles with initial monomer composition of 25% oNB-Glu and 75% Bn-Cys (top) and the images of the nanoparticle bulk solution at each time point (bottom).

[1] Y. V. Il'ichev, J. Wirz. Rearrangements of 2-Nitrobenzyl Compounds. 1. Potential Energy Surface of 2-Nitrotoluene and Its Isomers Explored with ab Initio and Density Functional Theory Methods**.** *The Journal of Physical Chemistry A* **2000** *104*, 7856-7870.
